# The conundrum of species delimitation: a genomic perspective on a mitogenetically super-variable butterfly

**DOI:** 10.1098/rspb.2019.1311

**Published:** 2019-09-18

**Authors:** Vlad Dincă, Kyung Min Lee, Roger Vila, Marko Mutanen

**Affiliations:** 1 Department of Ecology and Genetics, University of Oulu, PO Box 3000, 90014 Oulu, Finland; 2 Institut de Biologia Evolutiva (CSIC-Universitat Pompeu Fabra), Passeig Marítim de la Barceloneta, 37, 08003 Barcelona, Spain

**Keywords:** genomics, Lepidoptera, mitochondrial DNA, double-digest RAD sequencing, speciation, *Wolbachia*

## Abstract

The Palaearctic butterfly *Melitaea didyma* stands out as one of the most striking cases of intraspecific genetic differentiation detected in Lepidoptera: 11 partially sympatric mitochondrial lineages have been reported, displaying levels of divergence of up to 7.4%. To better understand the evolutionary processes underlying the diversity observed in mtDNA, we compared mtDNA and genome-wide SNP data using double-digest restriction site-associated DNA sequencing (ddRADseq) results from 93 specimens of *M. didyma* ranging from Morocco to eastern Kazakhstan. We found that, between ddRADseq and mtDNA results, there is a match only in populations that probably remained allopatric for long periods of time. Other mtDNA lineages may have resulted from introgression events and were probably affected by *Wolbachia* infection. The five main ddRADseq clades supported by STRUCTURE were parapatric or allopatric and showed high pairwise *F*_ST_ values, but some were also estimated to display various levels of gene flow. *Melitaea didyma* represents one of the first cases of deep mtDNA splits among European butterflies assessed by a genome-wide DNA analysis and reveals that the interpretation of patterns remains challenging even when a high amount of genomic data is available. These findings actualize the ongoing debate of species delimitation in allopatry, an issue probably of relevance to a significant proportion of global biodiversity.

## 1. Background

The study of global biodiversity is one of the fundamental commissions of biologists, but this task is also one of the most challenging due to the diversity of life on Earth and the resources needed to document it accurately. The species is the fundamental unit used to describe biodiversity and is a central concept in most studies on ecology and evolution, as well as nature conservation [1]. However, our knowledge of species diversity and distribution is far from complete and even estimates of global species numbers vary widely [2].

Biodiversity research is undergoing major progress due to the increasing use of molecular data that adds the genetic dimension to previous mostly morphological and/or ecological information. DNA barcoding—that is, the use of sequence variation in a short, standardized DNA marker to assign specimens to species [3]—has gained momentum and is arguably leading DNA-based efforts to assess global biodiversity. For animals, DNA barcoding relies on a part of the mitochondrial gene cytochrome *c* oxidase I (COI), which provides practical advantages in terms of sequence variation, DNA sequencing success and costs [3]. Its wide-scale use has led to the assembly of increasingly large DNA barcode libraries for various groups of organisms (e.g. [4–6]). Such relatively intensive screening has also revealed unexpected levels of intraspecific genetic differentiation in mtDNA in numerous species, even in well-studied taxonomic groups such as birds (e.g. [7,8]) and butterflies (e.g. [9,10]). In the latter, a recent study focused on Europe found that, while the majority of species displayed relatively low intraspecific divergence, 27.7% of 299 species DNA barcoded showed multiple evolutionarily significant units (ESU). Such studies help to set the standard for what divergence within and between species empirically is, and how generally this corresponds well to species boundaries, against which the outliers can be seen. These findings have also increased awareness towards a new layer of biodiversity represented by cryptic species (morphologically similar species that have been overlooked by scientists), which present new challenges to the study of biodiversity and to conservation efforts [11,12].

However, although DNA barcodes suggest the presence of a higher fraction of cryptic biodiversity than previously thought, conclusions cannot be drawn based on a single DNA marker. Yet the vast majority of studies investigating cases of deep intraspecific divergence in mtDNA used only a very small number of nuclear DNA markers based on Sanger sequencing. For example, for a highly diverse group such as butterflies, to our knowledge, only two recent studies addressed the issue using a genomic approach [13,14]. This underlines the need for further study to assess the significance of deep intraspecific DNA barcode splits and their implications for the global study of biodiversity.

The butterfly *Melitaea didyma* represents one of the most striking cases of mitochondrial DNA (mtDNA) divergence in Palaearctic butterflies. This species is part of the so-called *M. didyma* complex, in which no less than 23 highly diverged haplogroups have been reported [15]. Even for *M. didyma* alone, 11 partially sympatric mitochondrial lineages have been detected, displaying levels of mitochondrial DNA (mtDNA) divergence of up to 7.4% [15]. The apparent lack of morphological and ecological differentiation, as well as the very limited variability in chromosome number (*n* = 27–28), led to the conclusion that these 11 mtDNA lineages represent a case of extreme intraspecific mtDNA variability [15], but previous analyses did not include any nuclear DNA data.

In this study, we used a dataset of 93 specimens of *M. didyma sensu* *stricto* sampled mainly across Europe and North Africa, and directly compared results based on mtDNA and double-digest restriction site-associated DNA sequencing (ddRADseq) [16], a high-throughput sequencing technique that allows the recovery of thousands of loci across the nuclear genome. The 93 specimens were also screened for the presence of the maternally inherited bacterium *Wolbachia*. Using *M. didyma* as model, our goals were (1) to compare the evolutionary histories of mitochondrial and nuclear DNA and (2) to better understand evolutionary processes underlying deep mtDNA intraspecific splits and their potential to highlight cryptic diversity.

## 2. Methods

Methods are described in more detail in the electronic supplementary material.

### (a) Dataset used for molecular analyses

The core dataset was based on 93 specimens of *M. didyma* for which both COI sequences and ddRADseq data were obtained (electronic supplementary material, tables S1–S3). To this dataset, we added two specimens as outgroup taxa (*Melitaea trivia* and *Melitaea deione*) [17]. We followed [15] to assign the 93 specimens to mtDNA lineages (electronic supplementary material, figure S1). For this purpose, we assembled a dataset of 347 COI sequences obtained by combining the 93 COI sequences with data used by two recent studies focused on the *M. didyma* complex [15,18].

### (b) Mitochondrial DNA sequencing and analysis

COI sequences generated for this study were obtained using standard procedures (electronic supplementary material, table S4).

Phylogenetic relationships for the full dataset (347 COI sequences) were inferred using Bayesian inference (BI) through the CIPRES Science Gateway [19]. Both BI analyses and the estimation of node ages (based on published molecular clocks of 1.5% and 2.3% uncorrected pairwise distance per million years), see the electronic supplementary material for details) were run in BEAST 1.8.0 [20].

For the core dataset of 93 *M. didyma* COI sequences (and two outgroup samples; i.e. those specimens for which ddRADseq data were also available; electronic supplementary material, tables S1–S3), phylogenetic relationships were inferred using maximum likelihood (ML), to directly compare results with ML analyses based on ddRADseq data. The COI ML tree was inferred in RAxML v.8.2.0 [21] with bootstrap support estimated by a 1000 replicates rapid-bootstrap analysis from the unpartitioned GTR + CAT model.

### (c) ddRADseq library preparation and bioinformatics

Genomic DNA (gDNA) was extracted from one or two legs using the DNeasy Blood & Tissue Kit (Qiagen). To reach sufficient gDNA quantity and quality, whole genome amplification was performed using REPLI-g Mini Kit (Qiagen) due to its low concentrations of gDNA in the original extracts. The ddRADseq library was implemented following protocols described in [22] with an exception: the size distribution and concentration of the pools were measured with Bioanalyzer (Agilent Technologies).

Raw paired-end reads were demultiplexed with no mismatches tolerated using their unique barcode and adapter sequences using *ipyrad* v. 0.7.23 [23]. The demultiplexed paired-reads were run through PEAR [24] using default setting to merge overlapping reads, and input into the *ipyrad* pipeline. All *ipyrad* defaults were used, with the following exceptions: the minimum depth at which majority rule base calls are made was set to 3, the cluster threshold (*c*) was set to 0.90, the minimum number of samples (*m*) that must have data at a given locus for it to be retained was set to 4, 20, 30, 60 and 70, and the assembly method was set to *de novo*, *de novo*–reference and reference for independent testing. We used the *Melitaea cinxia* mitochondrion genome (GenBank accession CM002851) and whole-genome sequences (GCA_00071638) as references for the reference assembly. We also compiled a dataset of biallelic, unlinked SNPs by extracting a single SNP from each locus. The dataset of unlinked SNPs generated from the *ipyrad* datasets run with *c* 0.90 and *m* 20 was analysed using STRUCTURE and SNAPP.

### (d) Phylogenetic analysis of ddRADseq data

To study the phylogenetic relationships among taxa and to test the validity of prevailing species hypotheses, we conducted ML analyses inferred in RAxML v. 8.2.0 [21] for both concatenated and SNP RAD data.

The unlinked SNP datasets for species tree construction were imported into BEAUti, where the data were prepared for analyses with the SNAPP v. 1.1.16 plugin [25] in BEAST v. 2.1.3 [26]. We visualized the posterior distribution of species trees produced using DensiTree v. 2.2.1 [27].

### (e) Population structure and admixture

We inferred population clustering with admixture from SNP frequency data to visualize genomic variation between individuals with STRUCTURE [28].

FineRADstructure was used to investigate the genetic structure at population level within the *M. didyma* complex [29]. The package includes RADpainter, a program designed to infer the co-ancestry matrix and estimate the number of populations within the dataset.

*TreeMix* was used to identify patterns of divergence and admixtures, testing for migration events ranging from one to five [30]. This analysis was applied to a subset of 27 specimens that were also used for D-statistics, due to computational limitations (electronic supplementary material, table S2).

We used four-taxon D-statistics [31] to distinguish introgression from incomplete lineage sorting. All D-statistics were calculated in pyRAD v. 3.0.64 [32]. In order to run interactive data analysis, the Python Jupyter notebooks (https://jupyter.org) were used. The python script that we applied for D-statistics has been uploaded and are available from the Dryad Digital Repository: https://doi.org/10.5061/dryad.b883mf8 [33].

Pairwise *F*_ST_ values were calculated using Arlequin v.3.5 [34] and the proportion of missing data was calculated using Mesquite [35].

### (f) Coalescent-based species delimitation with Bayes factors

We performed Bayes factor species delimitation using the BFD* method [36], as implemented in SNAPP [25], based on a subset of specimens assuming five and eight taxa, respectively (electronic supplementary material, table S2). We assessed the strength of support of alternative species delimitation models following the scale of [37].

### (g) *Wolbachia* infection analyses

All 95 specimens for which COI and ddRADseq data were available were surveyed for the presence of the bacterium *Wolbachia* (electronic supplementary material, table S1).

The presence of *Wolbachia* was tested using PCR and sequencing primers specific to *Wolbachia* genes wsp and ftsZ (electronic supplementary material, table S4), which are extensively used to detect *Wolbachia* infection in a wide array of insects [38].

## 3. Results

### (a) Mitochondrial DNA

**Figure 1.**
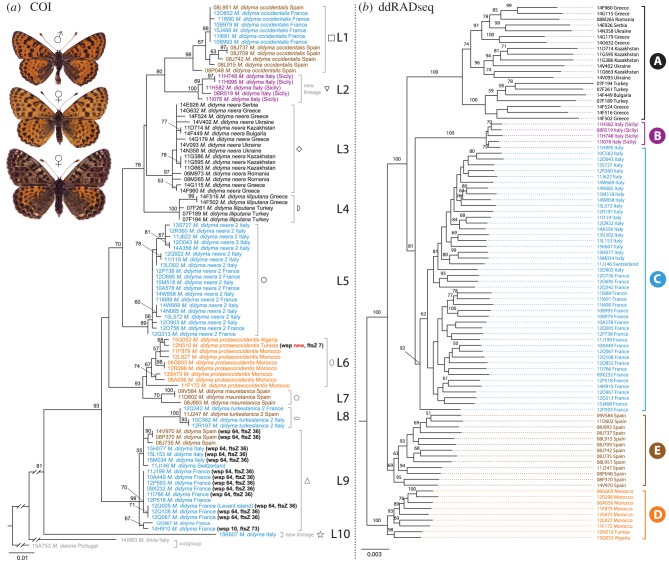
Maximum-likelihood (ML) trees of *Melitaea didyma*. (*a*) ML tree based on COI sequences. (*b*) ML tree inferred from the genome-wide data matrix using the *de novo*–reference assembly method (mitochondrial reads subtracted). Bootstrap values (1000 replicates) are indicated near the nodes. Branch lengths are proportional to the number of substitutions per site. Symbols used for the 10 COI lineages correspond to those used in figure 2. Colours used for COI sequences match the clade assignment based on ddRADseq data. For samples infected by *Wolbachia*, wsp and ftsZ alleles are indicated in (*a*). (Online version in colour.)

The 93 specimens of *M. didyma* used in the comparison between mtDNA and ddRADseq formed ten COI lineages (L1–L10; figure 1*a*; electronic supplementary material, figure S1). Eight of these lineages were assigned and named following [15] (electronic supplementary material, figure S1 and table S1), while two are reported here for the first time (L2, Sicily; and L10, a single specimen from north-western Italy). In the ML tree (figure 1*a*) the monophyly of the analysed samples of *M. didyma* was relatively well supported (bootstrap support 81). Most lineages were well supported, with the exception of L6 (bootstrap support under 50) and L9, the latter having been recovered as paraphyletic with respect to L8 as defined by Pazhenkova *et al*. [15]. The Bayesian analysis (electronic supplementary material, figure S1) recovered similar patterns, again not supporting the monophyly of L6 and L9. Furthermore, in this analysis, the addition of other species of the *M. didyma* complex broke the monophyly of *M. didyma*.

**Figure 2.**
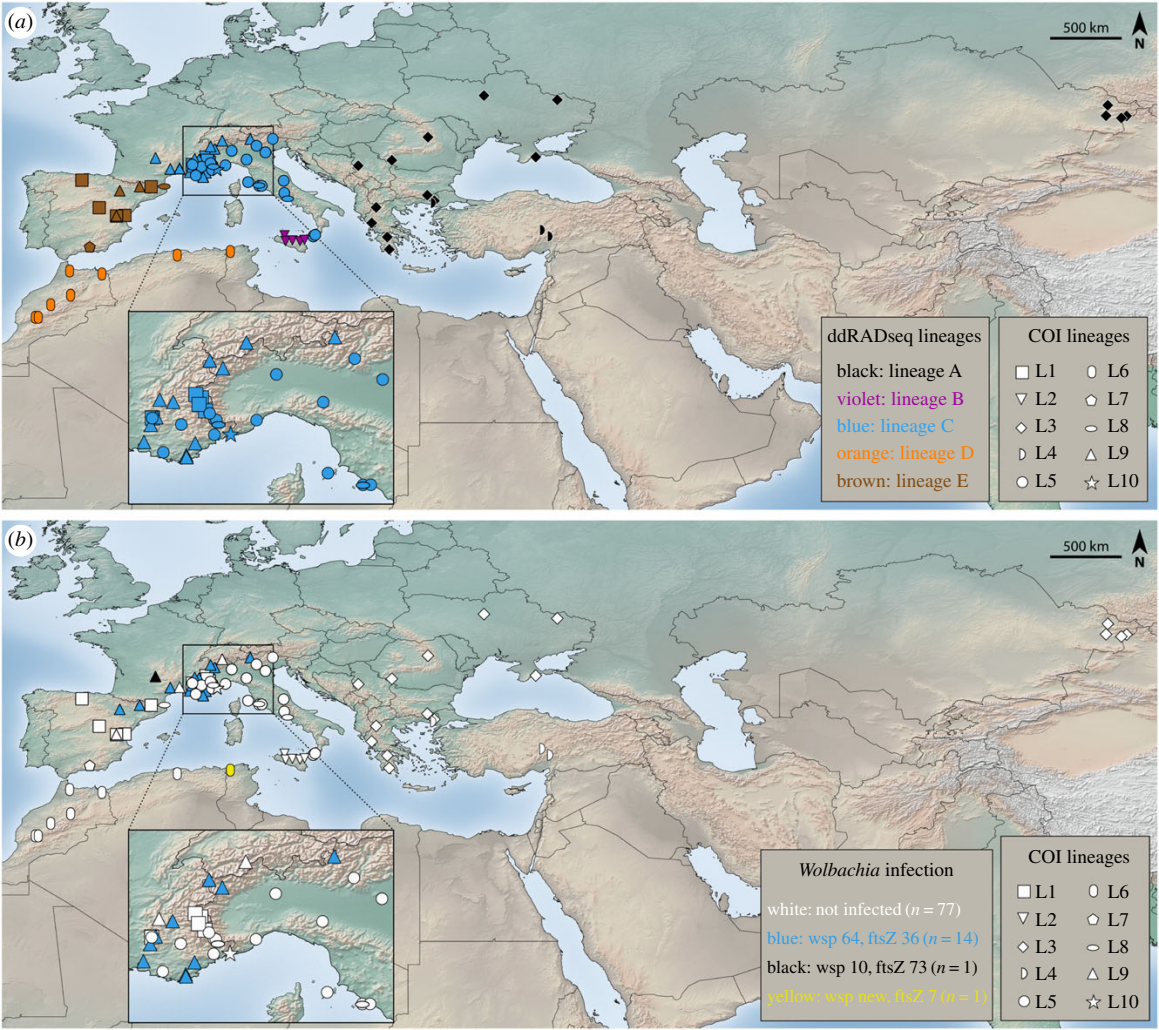
Geographical distribution (*a*) of ddRADseq and COI lineages of *Melitaea didyma* and (*b*) of *Wolbachia* infection. In (*a*), COI lineages are indicated by symbols and ddRADseq lineages by different colours. In (*b*), COI lineages are indicated by symbols and *Wolbachia* strains by different colours. Colours and symbols match those used in figure 1. (Online version in colour.)

The distribution of the 10 lineages is complex and it involves both allopatry and sympatry (figure 2). The Sicilian (L2) and North African (L6) lineages are the ones most clearly separated geographically, while cases of sympatry involve various lineages in Spain, France and Italy (figure 2; electronic supplementary material, table S1). Levels of divergence between lineages ranged from 1.2% to 7.5% minimum uncorrected p-distance (electronic supplementary material, table S5). L10, represented by a single Italian specimen, was most diverged from other lineages (5.6% minimum p-distance with respect to the nearest lineage). Even when L10 was not taken into account, minimum levels often exceeded 3.5% and even reached 5.0% (between L2 and L8; electronic supplementary material, table S5).

Excluding the highly diverged singleton belonging to L10, it appears that diversification within *M. didyma* started roughly 4.6 Ma (2.9 to 6.5 Ma, 95% CI; electronic supplementary material, figure S1).

### (b) ddRADseq data

We obtained 2.42 million reads per individual (electronic supplementary material, table S2). After filtering and clustering at 90% sequence similarity using the *de novo*–reference assembly method, we recovered 22 353 putative orthologous loci shared across more than four samples, for a total length of 3 489 654 base pairs (electronic supplementary material, table S3). These data include 143 201 SNPs, of which 46 371 are parsimony informative. For the reference assembly, an average of 238 205 reads per sample was mapped to the *Melitaea cinxia* genome (electronic supplementary material, table S2). After filtering, 18 636 clusters per sample were obtained, with an average of 43.5 cluster depth per sample. The final dataset from the reference assembly consisted of 14 525 recovered loci across more than four individuals (electronic supplementary material, table S3).

The ML analysis inferred from the genome-wide data matrix using the *de novo*–reference assembly method (figure 1*b*; electronic supplementary material, table S2) and species tree estimation analyses based on ddRAD data (electronic supplementary material, figure S2 and table S2) recovered five main lineages with allopatric distribution (figure 2*a*) within *M. didyma*. Minor differentiation was observed between other regions, such as between France and Italy in lineage C (figure 1*b*). Because the ML analysis did not include outgroup taxa, the ML tree was rooted based on the topology of the species tree, which clearly recovered the North African lineage (lineage D) as sister to the other four lineages (electronic supplementary material, figure S2). The species tree approach is less prone to misleading results because it incorporates uncertainty associated with gene trees (probability of unsorted ancestral polymorphism), nucleotide substitution model parameters and the coalescent process. The ML and species tree analyses (figure 1*b*; electronic supplementary material, figure S2) supported the Iberian lineage (lineage E) as sister to lineages A (eastern), B (Sicilian) and C (France–Italy). In the species tree, lineage C was weakly supported as sister to A and B, while the ML tree supported lineage A as sister to B and C.

ML trees inferred from the *de novo* assembly data matrix and the reference assembly data matrix against *Melitaea cinxia* genome recovered the same five clades mentioned above (electronic supplementary material, figure S3). As expected, the monophyly of some of the clades (e.g. lineages C and E) was affected when increasing the level of missing data due to the lack of phylogenetic signal, but the monophyly of lineages A, B and D was well supported even at 5% missing data (electronic supplementary material, figure S4). Lineage E generally displayed the highest level of missing data compared to the other lineages.

STRUCTURE indicated that five genetic clusters had the highest likelihood (electronic supplementary material, figure S5) and these clusters perfectly matched the ML analysis (figure 1*b*) and the FineRADstructure co-ancestry heat map (electronic supplementary material, figure S6).

The tree generated by FineRADstructure using SNPs indicated the presence of eight clusters, although the clustered co-ancestry heat map, suggested the existence of five main groups within *M. didyma* (electronic supplementary material, figure S6). This analysis revealed that the Sicilian population (lineage B) had the highest level of co-ancestry, while lineage C (France–Italy) displayed the lowest. The FineRADstructure result was corroborated by generally high (between 0.63 and 1) and significant pairwise *F*_ST_ values in all cases, with the exception of the comparison between lineages C (France–Italy) and E (Spain), where *F*_ST_ (0.65) was not significant (electronic supplementary material, table S6).

The analysis of patterns of divergence and admixture with *TreeMix* based on a subset of 27 specimens and allowing between one and five migration events, always estimated significant levels of gene flow from lineage B to D (Jackknife *p* = 0.00012), but also from A to C (*p* = 0.00030) (electronic supplementary material, figure S7). Tests of admixture using Patterson's D-statistics (based on the same 27 specimens used for *TreeMix*) confirmed the significant levels of gene flow recovered by *TreeMix* (B and D; A and C), but also estimated significant gene flow between D and A, as well as C and E (electronic supplementary material, table S8).

The Bayes factor species delimitation method using BFD* based on SNP data (see electronic supplementary material, table S2 for specimens used) recovered the five species hypothesis (corresponding to the five main lineages detected) as the most likely among nine competing species models. However, when eight species were assumed, this hypothesis was supported as the most likely (electronic supplementary material, table S7).

### (c) Incidence of *Wolbachia*

Fifteen (16%) of the 93 *M. didyma* specimens analysed were positive for infection by the bacterial endosymbiont *Wolbachia* (figures 1*a* and 2*b*; electronic supplementary material, table S1). Infected specimens displayed three combinations of wsp and ftsZ alleles suggesting the presence of three *Wolbachia* strains: wsp 64–ftsZ 36 was detected in 13 specimens, wsp 10–ftsZ 73 in one specimen and wsp ‘new’ (not assignable to allele using the *Wolbachia* MLST database)–ftsZ 7 in one specimen. Fourteen of the infected specimens belonged to mtDNA lineage L9 (involving Spanish, French and Italian specimens), while one specimen (alleles wsp new–ftsZ 7) belonged to mtDNA lineage L6 (Tunisian specimen; figures 1*a* and 2*b*).

## 4. Discussion

### (a) Phylogeography of *Melitaea didyma*

The most likely scenario consistent with our ddRADseq analyses suggests that diversification within *M. didyma* involved a first split separating the common ancestor into the African (clade D) and European populations (the rest of the clades) (figure 1*b*; electronic supplementary material, figure S2). Excluding the highly diverged L10 mtDNA lineage (represented by a single Italian specimen), the most recent common ancestor of the *M. didyma* samples analysed was dated roughly to 4.6 Ma (based on mtDNA; electronic supplementary material, figure S1), suggesting a long history of diversification spanning over several glacial cycles and a possible association with the Messinian salinity crisis that occurred about 5 Ma [39]. Nevertheless, this value needs to be taken with caution because of the technical limitations inherent in a molecular clock-based time estimate.

Only one lineage has been detected for North Africa both for the mitochondrial and nuclear data, indicating ongoing gene flow across the sampled area (from Morocco to Tunisia). Our analyses suggest that the first split within Europe separated the Iberian lineage from the rest, and was apparently generated by an expansion across the Pyrenees into central and eastern Europe (and further east into Asia), including the colonization of Sicily from the Italian mainland. The distribution of the five lineages recovered by the ddRAD data (figures 1*b* and 2*a*) suggest that differentiation occurred mainly through geographical isolation in refugia across several glacial cycles. Indeed, Iberia, the Italian peninsula and the Balkans are well-known European glacial refugia [40,41], and North Africa is increasingly recognized as a key region in shaping the biota of southern Europe [42].

Several of the clades are separated by significant geographical barriers that are likely to have played an important role in the formation of the detected patterns: the strait of Gibraltar (lineages D and E), the Pyrenees (lineages E and C) and the Messina strait (lineages C and B; figure 2*a*). Lineages C and A are currently separated by less obvious geographical barriers. It is possible that more extensive sampling will reveal contact zones among some of the lineages, most likely between C and A, and perhaps also between E and C. Several species of European butterflies display lineages apparently reflecting key refugia such as Iberia, Italy and the Balkans, but patterns vary and the prevalence of particular regions in harbouring endemic lineages has not been assessed yet across the entire butterfly fauna of the continent [10,41]. The Sicilian lineage (lineage B) of *M. didyma* reinforces observations of an unusually high number of endemic intraspecific genetic lineages on this island [43–45], despite the fact that the Messina strait separating Sicily from mainland Italy measures only 3 km at its narrowest point. The causes behind this phenomenon are not fully understood, but it appears that a combination of factors, such as reproductive interference, reduced dispersal, density-dependent phenomena and differences in climatic niches [43] may be at play. The Sicilian ddRAD lineage also displayed the highest level of co-ancestry (electronic supplementary material, figure S5) suggesting a population bottleneck (founder effect) associated with the colonization of the island.

### (b) Mito-nuclear discordance in *Melitaea didyma*

The mtDNA (COI) and the ddRAD datasets showed largely discordant patterns (figures 1 and 2). The only perfect match in terms of lineages recovered involved the Sicilian clade (clade B ddRAD, L2 mitochondrial). The North African lineage (clade D ddRAD, L6 mitochondrial) is also a match, but the monophyly of mtDNA L6 is actually not well supported (figure 1*a*; electronic supplementary material, figure S1). L6 was defined following [15] and represents a coherent geographical unit (North Africa), but mtDNA recovered it as related to L7 detected exclusively in southern Spain, the two taken together forming a well-supported clade (figure 1*a*, bootstrap = 93; electronic supplementary material, figure S1, posterior probability = 0.99). However, mtDNA L7 was recovered within ddRAD clade E (Iberia), together with all other Iberian specimens. This pattern suggests that mitochondrial introgression occurred at some point in the past from North Africa to southern Spain, but it is apparently presently not acting given that North African and southern Spain specimens do not share haplotypes.

Another partial match is represented by ddRAD clade A (eastern) and mtDNA L3 and L4, but the latter were not well supported as sister clades in the mtDNA analyses (figure 1*a*; electronic supplementary material, figure S1).

Clade C (France–Italy) included the entire mtDNA L5, as well as specimens from mtDNA L1, L8, L9 and the singleton representing L10. Since all L5 specimens belong to ddRAD clade C, L5 is probably the ancestral mitochondrial lineage for ddRAD clade C.

Clade E (Iberia) included all specimens from mtDNA L7, as well as specimens from L1, L8 and L9.

At least a part of these mismatches may have been facilitated by *Wolbachia*, which heavily infected mtDNA L9 (northern Spain, southern France, northern Italy; figures 1*a* and 2*b*). The maternally inherited bacterium *Wolbachia* is known for its potential to influence mtDNA genetic structure, particularly through asymmetric cytoplasmic incompatibility, when sperm from infected males cannot produce viable offspring with eggs of females that are not infected by the same *Wolbachia* strain [46]. Thus, *Wolbachia* infection can rapidly spread into a population, and because of maternal inheritance, can cause a selective sweep that favours the mitochondrial haplotype of the infected specimens. An increasing number of studies are reporting correlation between patterns of *Wolbachia* infection and mtDNA structure, and such cases have been reported for butterflies as well [45,47–50]. Furthermore, *Wolbachia* infections are dynamic and can be lost (e.g. [51,52]), a scenario that cannot be discarded for *M. didyma* as well, since some of its mtDNA lineages may have been infected in the past.

Mitochondrial L10, represented by an Italian singleton (sample 15K607) highly diverged from all lineages of *M. didyma* (figure 1*a*; electronic supplementary material, table S5), fell within clade C based on ddRAD data. This specimen was not infected by *Wolbachia* and, in the larger COI dataset (electronic supplementary material, figure S1), was recovered within a clade formed by other two specimens from Kazakhstan. This clade was phylogenetically more distant from *M. didyma* than other species of the *M. didyma* complex, which suggests introgression between relatively distant taxa. COI of sample 15K607 has been extracted and sequenced twice to discard the possibility of a contamination. The electropherograms were clean (i.e. without double peaks) and without stop codons, which could indicate the amplification of a pseudogene of mitochondrial origin in the nucleus (numt). Although numts can sometimes be notoriously difficult to detect [53], sample 15K607 is highly divergent (5.6%, electronic supplementary material, table S5) from the nearest conspecific, and it is likely that such a numt would have displayed at least some stop codons, deletions or insertions.

Overall, the mito-nuclear discordance detected in *M. didyma* is likely caused by a combination of introgression events, nuclear admixture and *Wolbachia* infection. The only cases where mtDNA was in complete agreement with the ddRAD patterns involved lineages that have likely remained allopatric (North Africa and Sicily) for a long time. Historical allopatry caused by glaciations may have also occurred in the three major European refugia, as suggested by the number of mitochondrial lineages. However, if these periods of isolation were shorter than in the case of North Africa and Sicily, the lack of reproductive isolation likely led to the nuclear admixture and sympatry of mitochondrial lineages. These findings suggest that long-term allopatry maintains genetic cohesion at parapatric boundaries (e.g. between the European refugia), which can be surpassed by non-neutral processes such as mitochondrial and/or *Wolbachia*-mediated introgression.

The higher number of mtDNA lineages, their complex distribution and relationships often not matching the ddRAD data, exemplify how mtDNA and nuclear DNA can have different evolutionary histories and call for caution when interpreting data-based solely on mtDNA. As a matter of fact, *M. didyma* is one of the few documented cases among European butterflies (e.g. genus *Lysandra* [54], *Iphiclides podalirius* and *I. feisthamelii* [55], *Melitaea phoebe* and *M. ornata* [56], genus *Brenthis* [57], *Thymelicus sylvestris* [14]) where mito-nuclear discordance is caused by biological processes, and not by operational factors (e.g. misidentifications, deficient taxonomy), although the latter have been shown to represent an important bias in European Lepidoptera [58].

### (c) Allopatry and species delimitation

The genetic patterns detected within *M. didyma* represent a prime example of the challenges associated with the delimitation of potential species in allopatry. The ddRAD analyses indicated the presence of five well-differentiated lineages (figure 1*a*; electronic supplementary material, figure S6) within *M. didyma*, although the Bayes factor species delimitation (BFD*) suggested an even higher structuring to eight lineages (electronic supplementary material, table S6 and supplementary methods for details).

Based on the current data, the five lineages are allopatric (figure 2*a*), although it is possible that further directed research may reveal areas of parapatry. However, given the nature of the current dataset (e.g. sampling across both sides of the Messina strait) and the presence of geographical barriers, we suspect it is unlikely that at least ddRAD clades B (Sicily) and D (North Africa) occur in sympatry with any other lineage.

Some of the clades were estimated to display significant levels of gene flow (electronic supplementary material, figure S7 and table S8), but D-statistics estimated more cases of introgression among lineages compared to *TreeMix*. However, we detected a limited power of the D-statistics analyses given the small fraction of bi-allelic sites that segregate between the focal populations. For this reason, the results should be interpreted with caution, especially when only a small number of loci were used for analyses.

It appears that clade C (France–Italy) is most actively involved in gene flow (between A and C according to *TreeMix*, and between C and E according to D-statistics), likely reflecting its geographical position between Iberia and clade E (eastern distribution) (figure 2*a*). The significant level of gene flow between lineages B (Sicily) and D (North Africa) may reflect the fact that hybridization and introgression have occurred in the past, but the variability of estimates (electronic supplementary material, figure S7 and table S8) also suggests that intrinsic limitations of the analyses (potentially also sensitive to sequence quality, levels of missing data, and sample size bias) should not be discarded. However, we are not aware of any study that specifically investigates the effect of various parameters on these methods when using large sets of genetic markers in cases of introgression/hybridization.

The allopatric ddRAD clades of *M. didyma* could be regarded as species under certain species concepts such as the phylogenetic species concept [59], but the application of this concept involves obvious risks of taxonomic inflation [60,61]. While the traditional use of relatively slow-evolving nuclear markers led to the general view that monophyly in nuclear trees is an indication of specific status (e.g. [55,56]), the nature of ddRAD data requires a change of paradigm because the resolution of such data is much higher than that of classical neutral nuclear markers. Given recombination, the resulting genetic distance is very sensitive to gene flow and is strongly influenced by isolation by distance. Virtually any case of allopatry (or even geographical discontinuity in sampling) will produce clades. Thus, while amount of data and resolution are no longer a problem with ddRAD data, caution about excessive taxonomic splitting is advisable.

*Melitaea didyma* is phenotypically very variable and apparently lacks clear morphological, ecological and chromosome number (*n* = 27–28) differences [15,62]. However, detailed morphological and/or ecological studies on the five nuclear DNA lineages reported here are lacking, while chromosome number counts were limited to three specimens and it is not impossible that future research may reveal certain differences. Furthermore, while taxonomists have traditionally used to assign much importance to morphological or ecological differences (even if sometimes fairly small), genetic differentiation based on thousands of loci from across the nuclear genome (as it is the case here) can hardly be regarded as less reliable compared to other characters.

The five ddRAD lineages detected clearly represent ESU and regarding them as distinct species may have important implications for species monitoring and conservation. For example, unlike the other large Mediterranean islands (Corsica, Sardinia, Crete), Sicily almost lacks endemic butterfly species. Thus, the Sicilian lineage of *M. didyma* would become the second butterfly species endemic to this island (together with *Hipparchia blachieri*) and its distribution and conservation status would need reassessment.

At the other extreme, regarding all lineages as conspecific may lead to an underestimation of the research and conservation value of various populations, given that species are the main target of monitoring and protection legislation (e.g. the EU Habitats Directive 92/43/EEC and most national laws).

Overall, the highly diverged but allopatric lineages of *M. didyma* illustrate a problem that is likely to be widespread across taxa since most species have a wide and uneven distribution, with isolated populations that are genetically differentiated to various degrees. This issue needs to be addressed in a practical way in order to accelerate the study of biodiversity and the solution will probably require that researchers reach a consensus regarding the operational criteria used for species delimitation. Genome-wide representations provided by RAD-sequencing approaches are not ideal in this respect. Although they are powerful in detecting genetic patterns (e.g. [63,64]), obtained genetic distances cannot be directly compared between datasets, because the proportion of missing data is associated with different types of loci retained [22,65]. Thus, it is hard to apply a general threshold to genetic distance obtained by ddRAD. However, comparisons of full genomes or techniques such as anchored hybrid enrichment [66] allow for direct comparisons at least across some taxonomic groups and can facilitate the inference of the best divergence thresholds for the delimitation of species.

An alternative to genetic distance (i.e. divergence) as an operational criterion to determine species status, is using values of geographical structuring or population differentiation (e.g. *F*_ST_, *D*_ST_ or *G*_ST_) or gene flow estimates between populations. These values are more suitable for comparisons across datasets but their interpretation is somewhat problematic: allopatry implies current lack of (or strongly reduced) gene flow, but the intensity and duration of historical gene flow may vary and it is hard to distinguish their contributions. Finally, not only conceptual but also methodological problems need to be resolved in order to compare and interpret gene flow estimates, as methods may be sensitive to dataset quality and used parameters, and results may be considerably different between different approaches.

*Melitaea didyma* highlights the potentially very different evolutionary histories of mitochondrial and nuclear DNA, as well as the need to further test and refine methods of gene flow and species inference. Although next-generation sequencing techniques can provide large amounts of genomic data, the conceptual problem of delimiting allopatric populations into species remains unchanged. This actualizes the call for a consensus on species boundaries in allopatry, when directly comparable genomic data may represent a practical solution to the complex reality generated by the process of speciation.

## Supplementary Material

Supplementary material

## Data accessibility

The *Melitaea* (COI) and *Wolbachia* (wsp and ftsZ) sequences generated for this study are available in GenBank (electronic supplementary material, table S1), and in the dataset DS-DIDYMA (doi:10.5883/DS-DIDYMA) from the Barcode of Life Data Systems (http://www.boldsystems.org/). The demultiplexed *Melitaea* fastq data are archived in the NCBI SRA: SRP144304. The python script applied for D-statistics has been uploaded and is available from the Dryad Digital Repository: https://doi.org/10.5061/dryad.b883mf8 [33].
