# Supplementary material for: The conundrum of species delimitation: a genomic perspective on a mitogenetically super-variable butterfly
Source: Proc Biol Sci. 2019 Sep 18;286(1911):20191311. doi: 10.1098/rspb.2019.1311 (PMC6784721; doi:10.1098/rspb.2019.1311)
Supplement: Supplementary material [file rspb20191311supp1.pdf]

Proceedings of the Royal Society B

Supplementary Material

The conundrum of species delimitation: a genomic perspective on a mitogenetically super-variable butterfly

Vlad Dincă, Kyung Min Lee, Roger Vila, Marko Mutanen

DOI: 10.1098/rspb.2019.1311

Material and Methods

Dataset used for molecular analyses

The core dataset was based on 93 specimens of *M. didyma* for which both COI sequences and ddRADseq data were obtained (Tables S1-S3). All ddRADseq data, as well as 77 COI sequences, were generated for this study. To this dataset we added two specimens as outgroup (*Melitaea trivia* and *Melitaea deione*) (Leneveu et al. 2009). We followed Pazhenkova & Lukhtanov (2016) to assign the 93 specimens to mtDNA lineages (Fig. S1). For this purpose, as well as to obtain rough estimates of divergence events, we assembled a dataset of 347 COI sequences obtained by combining the 93 COI sequences with data used by two recent studies focused on the *M. didyma* complex (Pazhenkova et al. 2015, Pazhenkova & Lukhtanov 2016). These studies also incorporated sequences originating from Wahlberg & Zimmermann (2000), Vila & Bjorklund (2004), Leneveu et al. (2009), Hausmann et al. (2011), Ashfaq et al. (2013) and Dincă et al. (2011, 2015). Sequence FJ663794 was not used because of the following discrepancy: our preliminary analyses showed that it clusters with taxon *sutschana*, but it was recovered within *latonigena* by Pazhenkova & Lukhtanov (2016).

Mitochondrial DNA sequencing

Thirty-six of the COI sequences generated for this study were obtained at the Butterfly Diversity and Evolution Lab of the Institut de Biologia Evolutiva (CSIC-UPF), Barcelona, Spain. In this case, total genomic DNA was extracted using Chelex 100 resin, 100–200 mesh, sodium form (Biorad), under the following protocol: one leg was removed and introduced into 100 µl of Chelex 10% and 5 µl of Proteinase K (20 mg/ml) were added. The samples were incubated overnight at 55ºC and were subsequently incubated at 100ºC for 15 minutes. Samples were then centrifuged for 10 s at 3000 rpm. A 658-bp fragment near the 5´ end of the mitochondrial gene COI was amplified by polymerase chain reaction (PCR) (primers used and PCR protocol available in Table S4). PCR products were purified and sequenced by Macrogen Inc.

The remaining sequences were generated at the Biodiversity Institute of Ontario, Canada following standard protocols for DNA barcoding (deWaard et al. 2008), and DNA sequencing was performed on an ABI 3730XL capillary sequencer (Applied Biosystems).
Sequences were edited in CodonCode Aligner 3.0 or in GENEIOUS PRO 6.1.8 (Biomatters, http://www.geneious.com/) and assembled using the latter.
The 79 COI sequences generated for this study (77 *M. didyma* plus two outgroup specimens) are available in GenBank (see Table S1 for accession numbers), and are also publicly available in the dataset DS-DIDYMA (dx.doi.org/10.5883/DS-DIDYMA) from the Barcode of Life Data Systems (http://www.boldsystems.org/).

Analyses of mitochondrial DNA sequences

Phylogenetic relationships for the full dataset (347 COI sequences) were inferred using Bayesian inference (BI) through the CIPRES Science Gateway (Miller et al. 2010). Both BI analyses and the estimation of node ages were run in BEAST 1.8.0 (Drummond & Rambaut 2007). The GTR + I + G substitution model was chosen according to the value of the Akaike information criterion (AIC) obtained in JMODELTEST 2.1.3 (Darriba et al. 2012). Base frequencies were estimated, six gamma rate categories were selected and a randomly generated initial tree was used.
Rough estimates of node ages were obtained by applying two molecular clocks with: 1.5% uncorrected pairwise distance per million years estimated for various invertebrates (Quek et al. 2004), and 2.3% estimated for the entire mitochondrial genome of several arthropods (Brower 1994). A lognormal relaxed clock and a normal prior distribution were used, centred on the mean between the two substitution rates, and the standard deviation was tuned so that the 95% confidence interval of the posterior density coincided with the 1.5% and 2.3% rates, respectively. Parameters were estimated using two independent runs of 40 million generations each, and convergence was checked using the program TRACER 1.6.

For the core dataset of 93 *M. didyma* COI sequences (and two outgroup samples) (i.e. those specimens for which ddRADseq data were also available) (Tables S1-S3), phylogenetic relationships were inferred using maximum likelihood (ML), to directly compare results with ML analyses based on ddRADseq data. The COI ML tree was inferred in RAxML v.8.2.0 (Stamatakis 2014) with bootstrap support estimated by a 1,000 replicates rapid-bootstrap analysis from the unpartitioned GTR+CAT model. We visualized the resulting phylogeny and assessed bootstrap support using FigTree v.1.4.2 (Rambaut 2015). The tree was rooted with *M. deione* (Leneveu et al. 2009).

ddRADseq library preparation and bioinformatics

In order to proceed with the ddRAD library preparation, genomic DNA (gDNA) was extracted from one or two legs using the DNeasy Blood & Tissue Kit (Qiagen). The quantity of gDNA extracts was checked using PicoGreen kit (Molecular Probes). To reach sufficient gDNA quantity and quality, whole genome amplification was performed using REPLI-g Mini Kit (Qiagen) due to its low concentrations of gDNA in the original extracts. The average original DNA extracts were 8.5 ng/µl and amplified up to 89.6 ng/µl (54.9 ng/µl on average) after whole genome amplification. The ddRADseq library was implemented following

protocols described in Lee *et al.* (2018) with an exception: the size distribution and concentration of the pools were measured with Bioanalyzer (Agilent Technologies). The demultiplexed *Melitaea* fastq data are archived in the NCBI SRA: SRP144304.
Raw paired-end reads were demultiplexed with no mismatches tolerated using their unique barcode and adapter sequences using *ipyrad* v.0.7.23 (Eaton and Overcast 2016). The quality of raw demultiplexed reads was checked with FastQC software (available at http://www.bioinformatics.babraham.ac.uk/projects/fastqc/). The demultiplexed paired-reads were run through PEAR (Zhang et al. 2014) using default setting to merge overlapping reads, and input into the *ipyrad* pipeline. All *ipyrad* defaults were used, with the following exceptions: the minimum depth at which majority rule base calls are made was set to 3, the cluster threshold was set to 0.90, the minimum number of samples that must have data at a given locus for it to be retained was set to 4, 20, 30, 60, and 70, and the assembly method was set to denovo, denovo–reference, and reference for independent testing. The ‘denovo’ method assembles sequences without any reference resources. Homology is inferred during alignment clustering by sequence similarity using the program *vsearch* (http://github.com/torognes/vsearch). The ‘denovo–reference’ method was used to exclude sequences that mapped to the *Melitaea cinxia* mitochondrion genome (GenBank accession CM002851). The ‘reference’ assembly method maps sequences to *M. cinxia* whole genome sequences (GenBank, GCA_00071638) with *BWA* using the default *bwa-mem* setting (Li 2013) based on 90% of sequence similarity.

Phylogenetic analysis of ddRADseq data

To study the phylogenetic relationships among taxa and to test the validity of prevailing species hypotheses, we conducted ML analyses. ML trees were inferred in RAxML v.8.2.0 (Stamatakis 2014) for the concatenated RAD data, with bootstrap support estimated by a 1,000 replicates rapid-bootstrap analysis from the unpartitioned GTR+CAT model. We visualized the resulting phylogeny and assessed bootstrap support using FigTree v.1.4.2 (Rambaut 2015).
The unlinked SNP datasets for species tree construction were imported into BEAUti (Bouckaert et al. 2014), where the data were prepared for analyses with the SNAPP v.1.1.16 plugin (Bryant et al. 2012) in BEAST v.2.1.3 (Bouckaert et al. 2014). The priors for forward (u) and reverse (v) mutation rates were set to be estimated and the remaining parameters were left at default values, e.g. species divergence rate $\lambda = 0.00765$, and θ defined by a γ prior with shape parameter $\alpha = 11.750$ and scale parameter $\beta = 109.73$. Runs were carried out for 10 million generations, sampling every 1,000 generations. The output was inspected with Tracer v.1.6 (Rambaut et al. 2013), ensuring stationarity and ESS > 200 for all parameters with a few exceptions for single theta values of internal branches in single replicates. We then visualized the posterior distribution of species trees produced using DensiTree v.2.2.1 (Bouckaert 2010).

Population structure and admixture

We inferred population clustering with admixture from SNP frequency data to better visualize genomic variation between individuals with STRUCTURE v.2.3.1 (Pritchard et al. 2000). Ten replicates were run with each value of *K* between 1 and 8. Each run had a burn-in

of 20K generations followed by 200K generations of sampling. Replicates were permuted using CLUMPP (Jakobsson and Rosenberg 2007) and the optimal *K* value inferred using StructureHarvest (Earl and VonHoldt 2012) according to the ad hoc Δ*K* statistics (Evanno et al. 2005), which is the second-order rate of change of the likelihood function. STRUCTURE results were visualized using DISTRUCT (Rosenberg 2004).

FineRADstructure was used to investigate the genetic structure at population level within the *M. didyma* complex (Malinsky et al. 2018). The package includes RADpainter, a program designed to infer the co-ancestry matrix and estimate the number of populations within the dataset. The input file used was an allele.loci matrix (20% of missing data) generated by *ipyrad* program. The allele data was converted using a python script available at https://github.com/edgardomortiz/fineRADstructure-tools (last accessed April 20, 2018). Then, the individuals were assigned to populations and the phylogenetic tree was built using the fineSTRUCTURE MCMC clustering algorithm.

TreeMix was used to identify patterns of divergence and admixtures, testing for migration events ranging from one to five including possible admixture with two closely related outgroup samples included in the analyses (Pickrell and Pritchard 2012). This method constructs a bifurcating tree of populations using 100 bootstrap replicates and it identifies potential episodes of gene flow from the residual covariance matrix. This analysis was applied to a subset of 27 specimens that were also used for D-statistics (see below). This subset consisted of five representative samples from each of the five lineages, as well as two outgroups (*Melitaea trivia* and *M. deione*), that were chosen in order to equalize the sample size based on the highest number of recovered loci in the final data matrix (Table S2).

We used four-taxon D-statistics (Durand et al. 2011) to distinguish introgression from incomplete lineage sortingbased on the same subset of 27 specimens used for *TreeMix*. The full dataset could not be used due to computational limitations. The test is based on the assumption of a true four-taxon asymmetric phylogeny (((P1, P2) P3,) O). All sites considered in the alignment of sequences from these taxa must be either mono or biallelic, with the outgroup defining the ancestral state 'A' relative to the derived state 'B'. If two alleles are present in a site, the possible combinations are ABBA and BABA. The D-statistics compares the occurrence of these two discordant site patterns, representing sites where an allele is derived in P3 relative to outgroup (O), and is derived in one but not both of the sister lineages P1 and P2. These discordant sites can arise through the sorting of ancestral polymorphisms. In absence of introgression, the frequencies for these two outcomes are expected to be equal. This finding would support incomplete lineage sorting (ILS) being responsible for barcode sharing, while deviation from it would support introgression (Durand et al. 2011). For the test, 1,000 bootstrap replicates were performed to measure the standard deviation of the D-statistics. Significance was evaluated by converting the Z-score (which represents the number of standard deviations from zero from D-statistics) into two tailed P-values, and using α=0.01 as a conservative cutoff for significance after correcting for multiple comparisons using Holm-Bonferroni correction. All D-statistics were calculated in pyRAD

v.3.0.64 (Eaton 2014). In order to run interactive data analysis, the Python Jupyter notebooks (https://jupyter.org) were used.
The python script that we applied for D-statistics has been uploaded and shared via Dryad (DOI: http://doi.org/10.5061/dryad.b883mf8).
Pairwise F_{ST} values were calculated using Arlequin v.3.5 (Excoffier and Lischer 2010). Statistical significance of the F_{ST} values was tested by permutation analysis with 1,000 permutations. The proportion of missing data was calculated using Mesquite (Maddison and Maddison 2017).

Coalescent-based species delimitation with Bayes factors
We performed Bayes factor species delimitation using the BFD* method (Leaché et al. 2014) based on a subset of specimens (see Table S2) plus 2 outgroup specimens, due to computational limitations. This method allows for the comparison of alternative species delimitation models in an explicit multispecies coalescent framework using genome-wide SNP data, implemented using SNAPP (Bryant et al. 2012). We tested nine (when assuming 5 taxa) and ten competing species models (when assuming 8 taxa) for *M. didyma*: 37 specimens (including 2 outgroup specimens) when assuming 5 taxa; 41 specimens (including 2 outgroup specimens) when assuming 8 taxa. The specimens were selected based on the highest number of recovered loci in the final data matrix. The full dataset could not be used due to computational limitations. For all species models, we conducted path sampling for a total of 24 steps (200,000 MCMC steps, 10,000 burn-in steps each) to calculate marginal likelihood estimates (MLE) for each competing model. Bayes factor (BF) support was compared between models to identify the best-supported species model. We assessed the strength of support of alternative species delimitation models following the scale of Kass & Raftery (1995).
When assuming 5 taxa, that hypothesis was best supported, but when assuming 8 taxa, that hypothesis was best supported (Table S6). The results should be interpreted with caution as there is recent evidence suggesting that hypotheses considering more species/lineages are better supported than those assuming less splitting (e.g. Sukumaran & Knowles 2017; O'Connell & Smith 2018). Only few studies have examined the effect of missing data when using BFD*, which does not accommodate missing data between assigned species. The inclusion of more loci, even at the expense of very high amounts of missing data, led to higher BF and better resolved species trees than datasets with less missing data but fewer loci. This is because less stringent filtering retains lineage-specific loci, which may help coalescent methods to better delimit lineages. Therefore, caution should be taken in interpreting BFD* results on the basis of different levels of missing data.

Wolbachia infection analyses
All 95 specimens for which COI and ddRADseq data were available were surveyed for the presence of the bacterium *Wolbachia* (Table S1).
The presence of *Wolbachia* was tested using PCR and sequencing primers specific to *Wolbachia* genes wsp and ftsZ, which are extensively used to detect *Wolbachia* infection in a wide array of insects (Baldo et al. 2006). Primers used and PCR protocols are available in Table S4.

Samples with amplicons of the expected size (as visualized on agarose gels) were scored as positive for *Wolbachia* and PCR products of all infected specimens were sequenced.

Sequences were then compared to existing records using the *Wolbachia* MLST Database (pubmlst.org/wolbachia/) in order to identify the sequence type for each gene locus. Sequences obtained during the screening are available in GenBank (see Table S1 for accession numbers) and in the dataset DS-DIDYMA (dx.doi.org/10.5883/DS-DIDYMA) from the Barcode of Life Data Systems (http://www.boldsystems.org/).

References

Ashfaq M, Akhtar S, Khan AM, Adamowicz SJ, Hebert PDN. 2013. DNA barcode analysis of butterfly species from Pakistan points towards regional endemism. *Molecular Ecology Resources* **13**, 832–843. (doi: 10.1111/1755-0998.12131)

Baldo L, Dunning Hotopp JC, Jolley KA, Bordenstein SR, Biber SA, Choudhury RR, Hayashi C, Maiden MC, Tettelin H, Werren JH. 2006. Multilocus Sequence Typing System for the Endosymbiont *Wolbachia pipientis. Applied and Environmental Microbiology* **72**(11), 7098-7110. (doi:10.1128/AEM.00731-0)

Bouckaert R. 2010. DensiTree: making sense of sets of phylogeneftic trees. *Bioinformatics*, **26**, 1372–1373.

Bouckaert R, Heled J, Kühnert D *et al.* 2014. BEAST 2: a software platform for Bayesian evolutionary analysis. *PLoS Computational Biology*, **10**, e1003537.

Bouckaert R, Heled J, Kühnert D, Vaughan T, Wu C-H, Xie D, Suchard MA, Rambaut A, Drummod AJ. 2014. BEAST 2: a software platform for Bayesian evolutionary analysis. *PLoS Computational Biology*, **10**, e1003537. (https://doi.org/10.1371/journal.pcbi.1003537)

Brower AVZ. 1994. Rapid morphological radiation and convergence among races of the butterfly *Heliconius erato* inferred from patterns of mitochondrial DNA evolution. *PNAS* **91**, 6491–6495. (https://doi.org/10.1073/pnas.91.14.6491)

Bryant D, Bouckaert R, Felsenstein J, Rosenberg NA, Roychoudhury A. 2012. Inferring species trees directly from biallelic genetic markers: Bypassing gene trees in a full coalescent analysis. *Molecular Biology and Evolution*, **29**, 1917–1932.

Darriba D, Taboada GL, Doallo R, Posada D. 2012. jModelTest 2: more models, new heuristics and parallel computing. *Nature Methods* **9**, 772. (doi:10.1038/nmeth.2109)

deWaard JR, Ivanova NV, Hajibabaei M, Hebert PDN. 2008. Assembling DNA Barcodes: Analytical Protocols. In *Methods in Molecular Biology: Environmental Genetics* (ed. Cristofre M.), pp. 275–293. Totowa, USA: Humana Press Inc.

Dincă V, Zakharov EV, Hebert PDN, Vila R. 2011. Complete DNA barcode reference library for a country's butterfly fauna reveals high performance for temperate Europe. *Proceedings of the Royal Society B* **278**, 347–355. (DOI: 10.1098/rspb.2010.1089)

Dincă V, Montagud S, Talavera G, Hernández-Roldán J, Munguira ML, García-Barros E, Hebert PDN, Vila R. 2015. DNA barcode reference library for Iberian butterflies enables a continental-scale preview of potential cryptic diversity. *Scientific Reports* **5**, 12395. (doi:10.1038/srep12395)

Drummond AJ, Rambaut A. 2007. BEAST: Bayesian evolutionary analysis by sampling trees. *BMC Evolutionary Biology* **7**, 214. (https://doi.org/10.1186/1471-2148-7-214)

Durand EY, Patterson N, Reich D, Slatkin M. 2011. Testing for ancient admixture between closely related populations. *Molecular Biology and Evolution* **28(8)**, 2239-2252. (https://doi.org/10.1093/molbev/msr048)

Earl DA, VonHoldt BM. 2012. STRUCTURE HARVESTER: a website and program for visualizing STRUCTURE output and implementing the Evanno method. *Conserv. Genet. Resour.* **4**, 359–361. (doi:10.1007/s12686-011-9548-7)

Eaton DAR, 2014. PyRAD: assembly of de novo RADseq loci for phylogenetic analyses. *Bioinformatics* **30(13)**, 1844–1849. (doi: 10.1093/bioinformatics/btu121)

Eaton DAR, Overcast I. 2016. ipyrad: interactive assembly and analysis of RADseq data sets. Available from: http://ipyrad.readthedocs.io/.

Evanno G, Regnaut S, Goudet J. 2005. Detecting the number of clusters of individuals using the software structure: a simulation study. *Mol. Ecol.* **14**, 2611–2620. (doi:10.1111/j.1365-294X.2005.02553.x)

Excoffier L, Lischer H. 2010. Arlequin suite ver 3.5: a new series of programs to perform population genetics analyses under Linux and Windows. *Molecular Ecology Resources*, **10**, 564–567. (doi: 10.1111/j.1755-0998.2010.02847.x.)

Hausmann A., Haszprunar G., Segerer AH, Speidel W, Behounek G, Hebert PDN. 2011. Now DNA-barcoded: the butterflies and larger moths of Germany. *Spixiana* **34(1)**, 47–58.

Jakobsson M, Rosenberg NA. 2007. CLUMPP: A cluster matching and permutation program for dealing with label switching and multimodality in analysis of population structure. *Bioinformatics* **23**, 1801–1806. (doi:10.1093/bioinformatics/btm233)

Kass R, Raftery A. 1995. Bayes factors. *Journal of the american statistical association* **90**, 773–795.

Lee KM, Kivelä SM, Ivanov V, Hausmann A, Kaila L, Wahlberg N, Mutanen M. 2018. Information Dropout Patterns in Restriction Site Associated DNA Phylogenomics and a Comparison with Multilocus Sanger Data in a Species-Rich Moth Genus. *Systematic Biology* **67(6)**, 925–939. (https://doi.org/10.1093/sysbio/syy029)

Leneveu J, Chichvarkhin A, Wahlberg N. 2009. Varying rates of diversification in the genus *Melitaea* (Lepidoptera: Nymphalidae) during the past 20 million years. *Biological Journal of the Linnean Society* **97**, 346–361. (doi: 10.1111/j.1095-8312.2009.01208.x)

Leaché AD, Fujita MK, Minin VN, Bouckaert RR. 2014. Species delimitation using genome-wide SNP Data. *Systematic Biology*, **63**, 534–542. (https://doi.org/10.1093/sysbio/syu018)

Li H. 2013. Aligning sequence reads, clone sequences and assembly contigs with BWA-MEM. *arXiv*, 1303.3997.

Maddison WP, Maddison DR. 2017. Mesquite: a modular system for evolutionary analysis. Version 3.2.

Malinsky M, Trucchi E, Lawson DJ, Falush D. 2018. RADpainter and fineRADstructure: Population Inference from RADseq Data. *Molecular Biology and Evolution* **35**, 1284–1290. (doi: 10.1093/molbev/msy023)

Miller MA, Pfeiffer W, Schwartz T. 2010. Creating the CIPRES Science Gateway for inference of large phylogenetic trees. In *Proceedings of the Gateway Computing*

Environments Workshop (GCE), New Orleans, LA, Nov. 14, 2010. Institute of Electrical and Electronics Engineers, pp 1–8. (doi: 10.1109/GCE.2010.5676129)

O'Connell KA, Smith EN. 2018. The effect of missing data on coalescent species delimitation and a taxonomic revision of whipsnakes (Colubridae: *Masticophis*). *Molecular Phylogenetics and Evolution* **127**, 356–366. (https://doi.org/10.1016/j.ympev.2018.03.018)

Pazhenkova EA, Zakharov EV, Lukhtanov VA. 2015. DNA barcoding reveals twelve lineages with properties of phylogenetic and biological species within *Melitaea didyma* sensu lato (Lepidoptera, Nymphalidae). *ZooKeys* **538**, 35–46. (https://doi.org/10.3897/zookeys.538.6605)

Pazhenkova EA, Lukhtanov VA. 2016. Chromosomal and mitochondrial diversity in *Melitaea didyma* complex (Lepidoptera, Nymphalidae): eleven deeply diverged DNA barcode groups in one non-monophyletic species? *Comparative Cytogenetics* **10(4)**, 697–717. (doi: 10.3897/CompCytogen.v10i4.11069)

Pickrell JK, Pritchard JK. 2012. Inference of population splits and mixtures from genome-wide allele frequency data. *PLoS Genetics*, **8**, e1002967. (https://doi.org/10.1371/journal.pgen.1002967)

Pritchard JK, Stephens M, Donnelly P. 2000. Inference of population structure using multilocus genotype data. *Genetics* **155**, 945–959. (doi:10.1111/j.1471-8286.2007.01758.x)

Quek SP, Davies SJ, Itino T, Pierce NE. 2004. Codiversification in an ant-plant mutualism: stem texture and the evolution of host use in *Crematogaster* (Formicidae: Myrmicinae) inhabitants of *Macaranga* (Euphorbiaceae). *Evolution* **58**, 554–570. (doi: 10.1111/j.0014-3820.2004.tb01678.x)

Rambaut A. 2015. FigTree, v1.4.2: Tree Figure Drawing Tool. Molecular evolution, phylogenetics and epidemiology. Available from: http://tree.bio.ed.ac.uk/software/figtree/.

Rambaut A, Suchard M., Drummond A. 2013. Tracer v. 1.6. Available from: http://tree.bio.ed.ac.uk/software/tracer/.

Rosenberg N. 2004. DISTRUCT: a program for the graphical display of population structure. *Mol. Ecol. Notes* **4**, 137–138. (doi:10.1046/j.1471-8286.2003.00566.x)

Stamatakis A. 2014. RAxML version 8: A tool for phylogenetic analysis and post-analysis of large phylogenies. *Bioinformatics* **30**, 1312–1313. (doi: 10.1093/bioinformatics/btu033)

Sukumaran J, Knowles LL. 2017. Multispecies coalescent delimits structure, not species. *PNAS* **114(7)**, 1607–1612. (https://doi.org/10.1073/pnas.1607921114)

Vila R, Bjorklund M. 2004. The utility of the neglected mitochondrial control region for evolutionary studies in Lepidoptera (Insecta). *Journal of Molecular Evolution* **58(3)**, 280–290. (doi: 10.1007/s00239-003-2550-2)

Wahlberg N, Zimmermann M. 2000. Pattern of phylogenetic relationships among members of the tribe Melitaeini (Lepidoptera: Nymphalidae) inferred from mitochondrial DNA sequences. *Cladistics* **16**, 347–363. (doi: 10.1006/clad.2000.0136)

Zhang J, Kobert K, Flouri T, Stamatakis A. 2014. PEAR: a fast and accurate Illumina Paired-End reAd mergeR. *Bioinformatics* **30**, 614–620. (doi: 10.1093/bioinformatics/btt593)

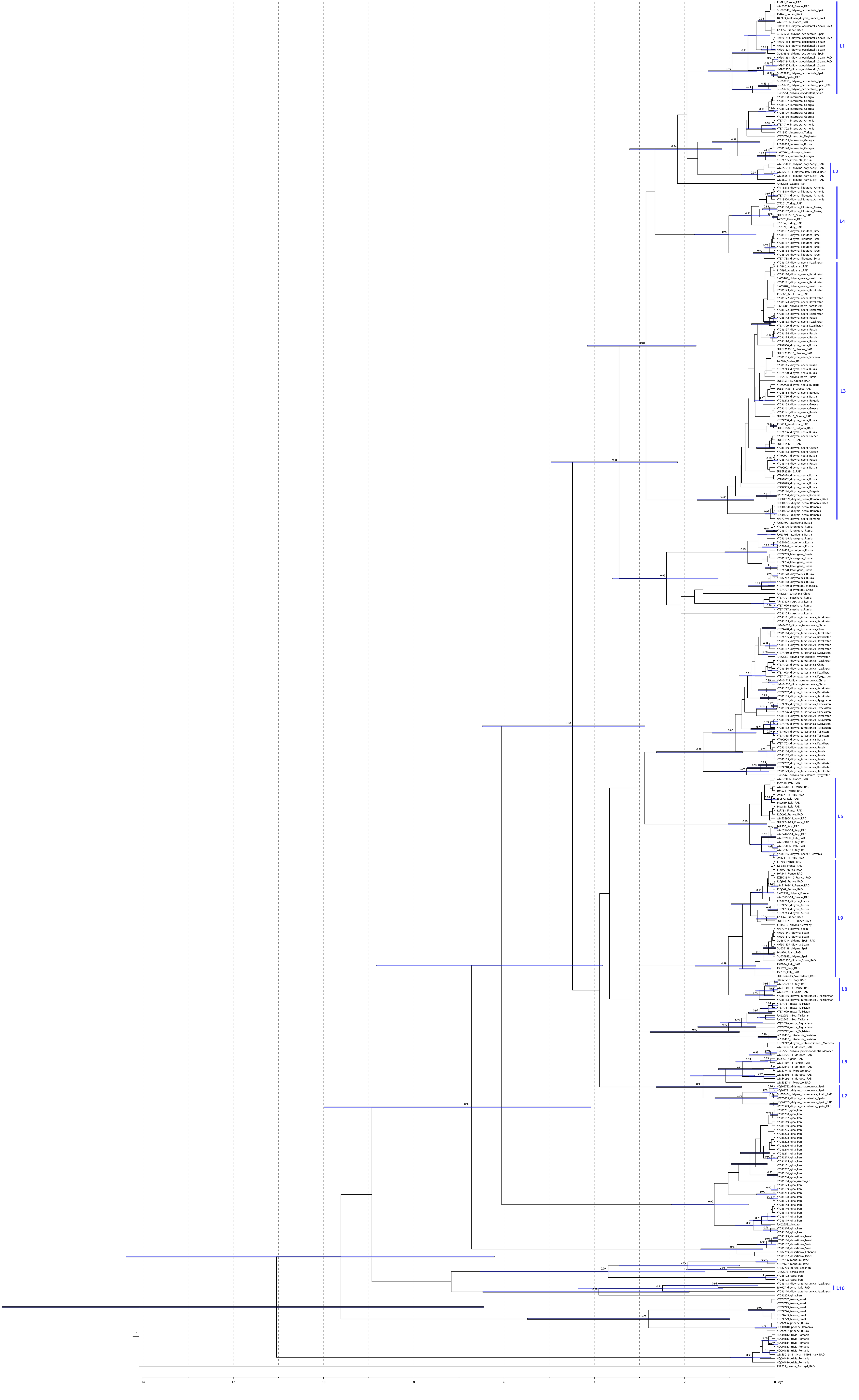

Fig. S1. Bayesian ultrametric tree for *Melitaea didyma* and related taxa based on cytochrome c oxidase subunit 1 sequences (numbers above recovered nodes, Bayesian posterior probabilities >0.7; node bars, 95% highest posterior density for age estimations; blue vertical bars, main COI lineages of *M. didyma* included in the comparison with ddRADseq data; specimens followed by "RAD" have been used for ddRADseq analyses).

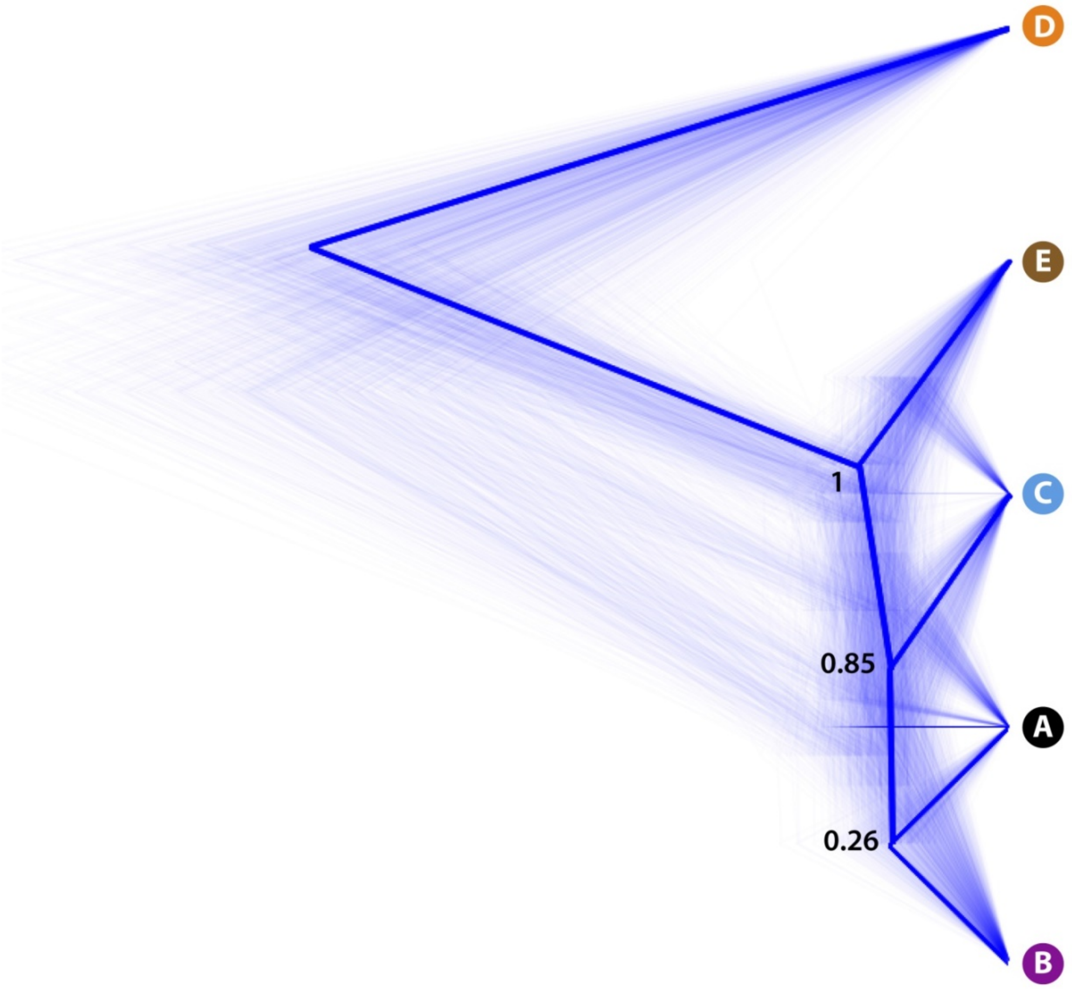

Fig. S2. DensiTree of the posterior distribution of SNAPP trees from 295 unlinked SNPs mined from ddRAD loci. The five species/clades were identified by the species delimitation analysis. The consensus is represented in thick blue lines within the species tree and the posterior probability is indicated for each node.

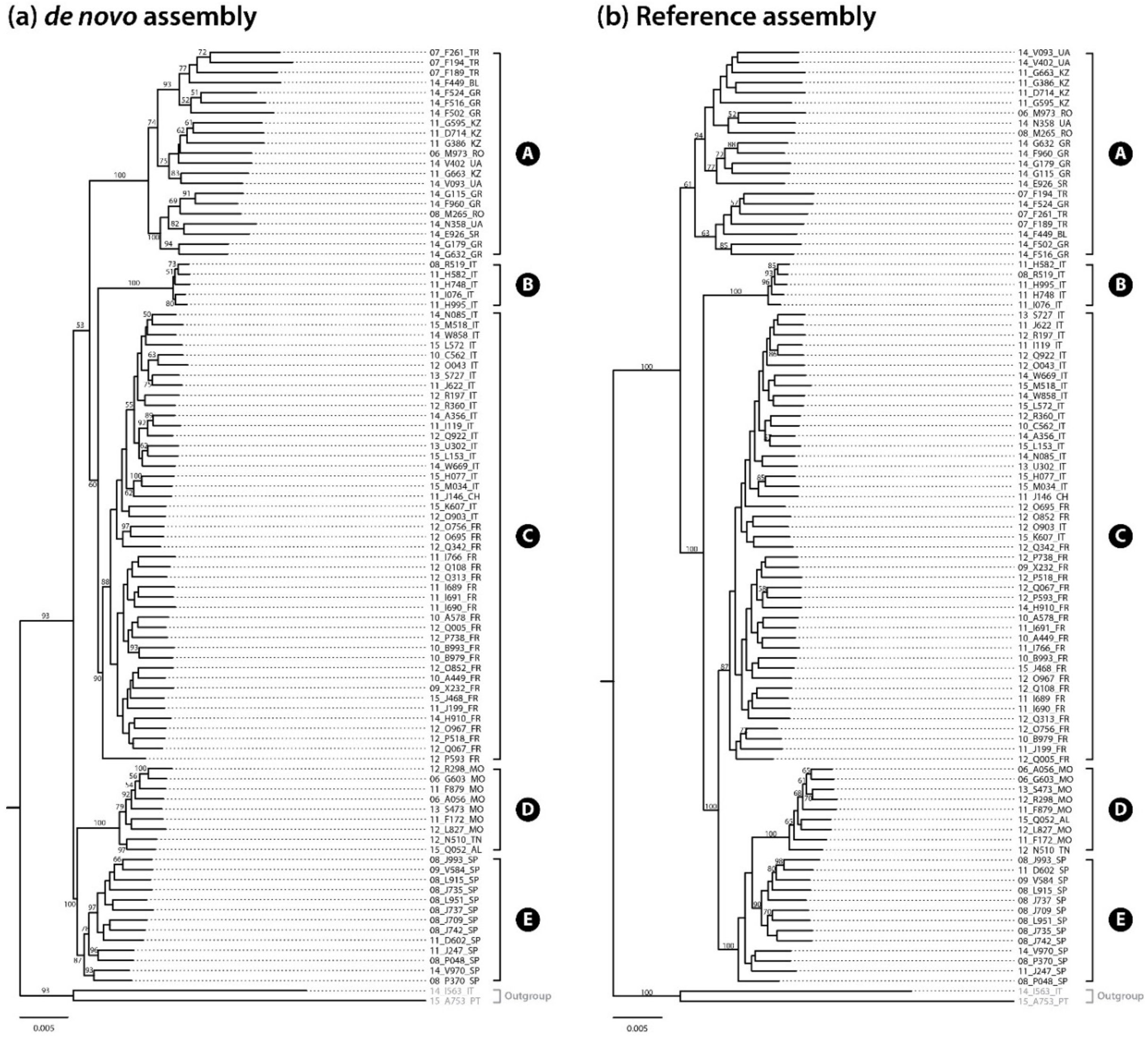

Fig. S3. ML trees inferred from **(a)** the *de novo* assembly data matrix and **(b)** the reference assembly data matrix against *Melitaea cinxia* genome (GCA_000716385) including two outgroup specimens. The phylogenetic trees were inferred with RAxML with 1,000 bootstrap replicates. Bootstrap values are indicated near branches. Only bootstrap values > 50% are shown. Branch lengths are proportional to the number of substitutions per site.

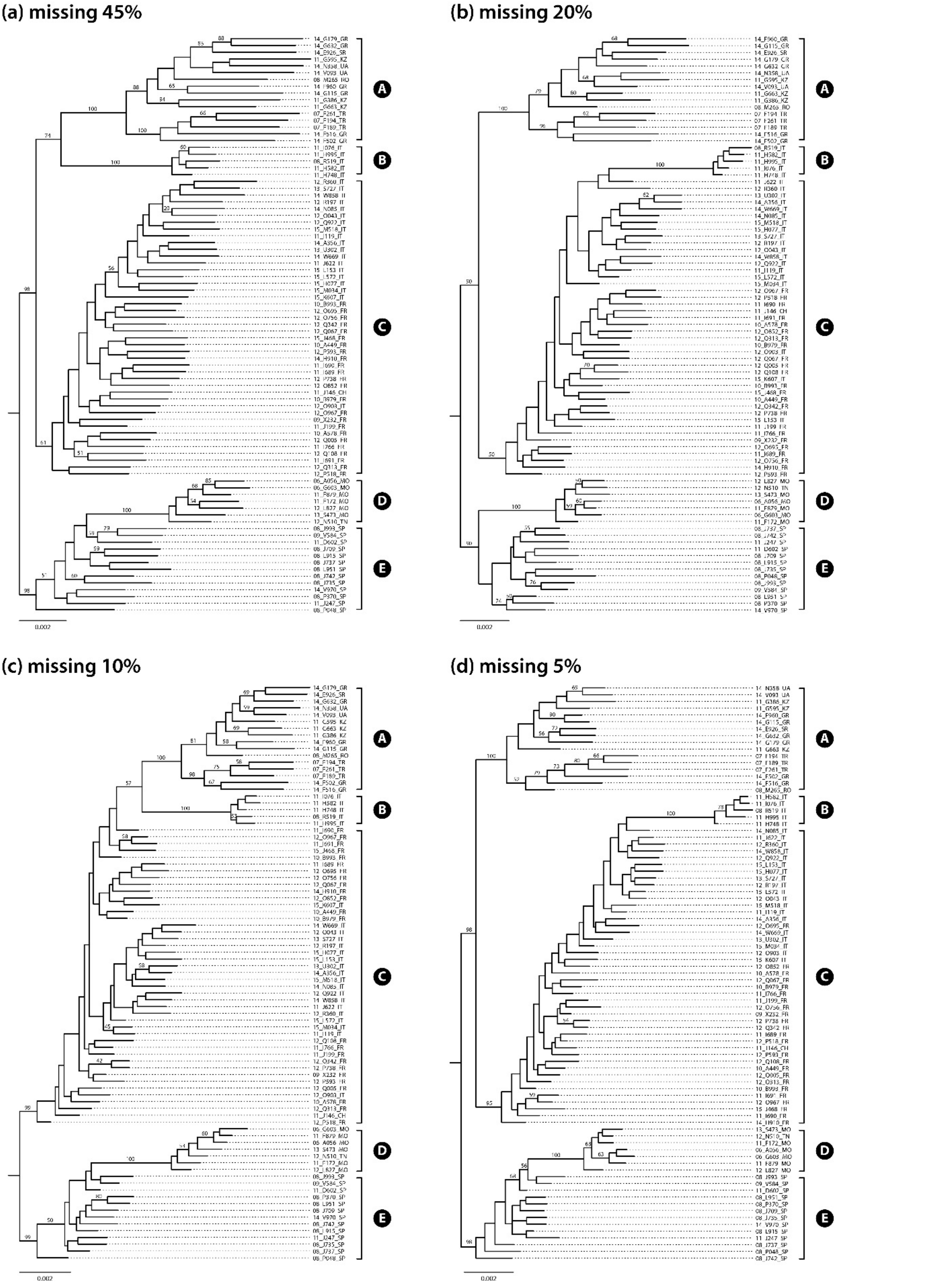

Fig. S4. ML trees based on **(a)** 45% of missing, **(b)** 20% of missing, **(c)** 10% of missing, and **(d)** 5% of missing ddRAD data.

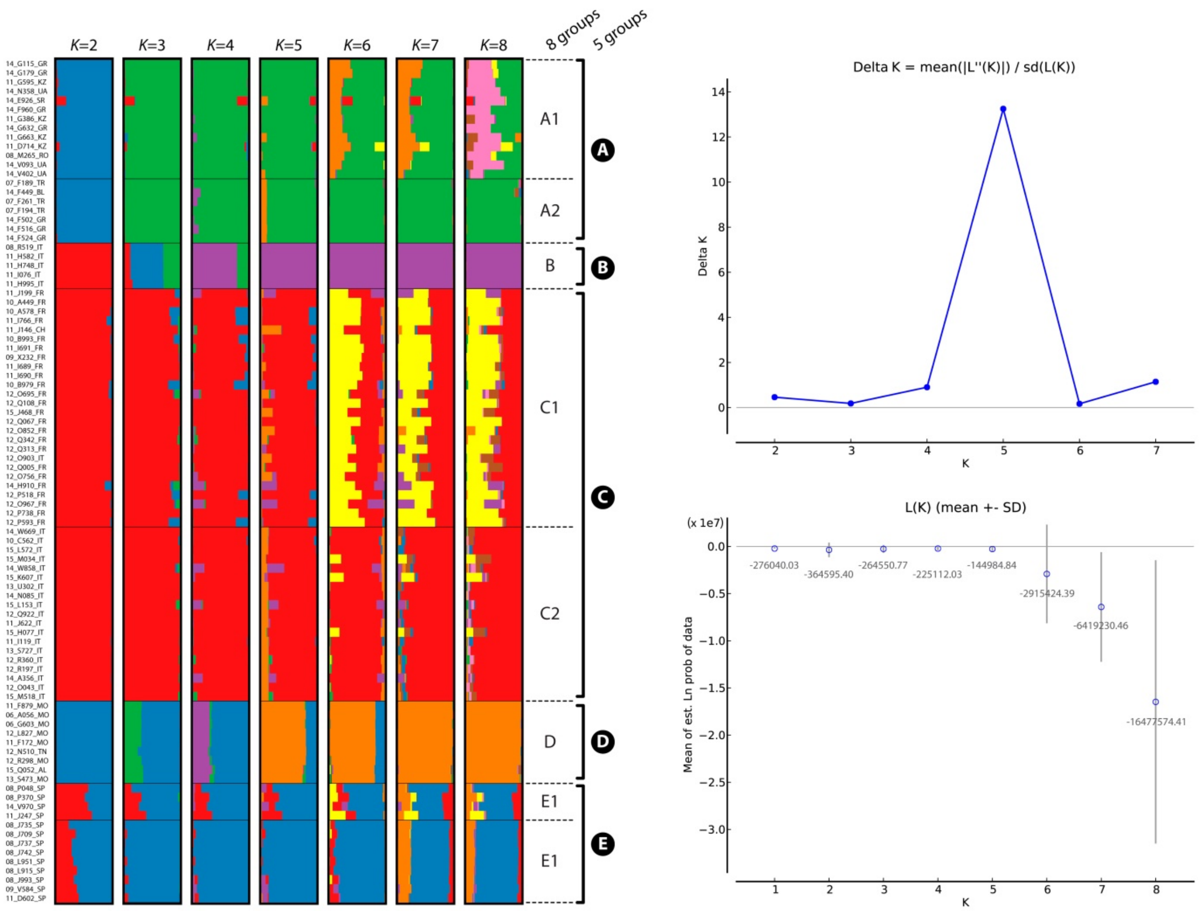

Fig. S5. Population genetic structure of the estimated delta K (ΔK) value for ten run replications of each from K=2 to K=8. The ΔK graph determined the maximum value at K=5. Mean probabilities lnP(K) and their standard deviation of posterior probability are shown. In the lower right panel, the numbers under the small circles represent mean estimated likelihood values for each K.

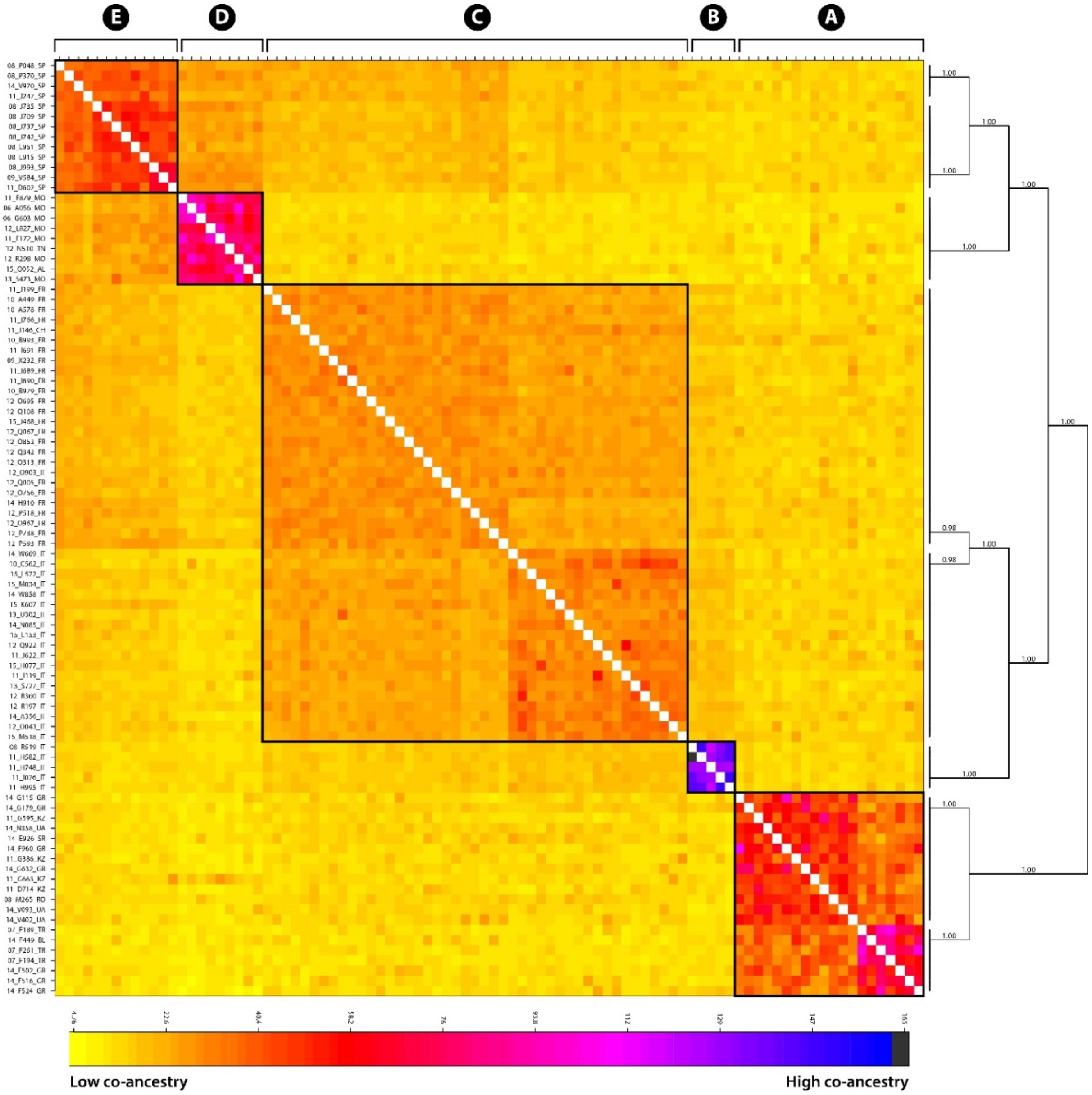

Fig. S6. Clustered FineRADstructure co-ancestry matrix for *M. didyma*. The highest levels of co-ancestry are evident among individuals from Sicily population (clade B), indicated by black, blue and purple colours. The lowest levels of co-ancestry sharing are indicated by yellow coloration. Individuals clustering into species/populations are indicated by clustering in the accompanying tree.

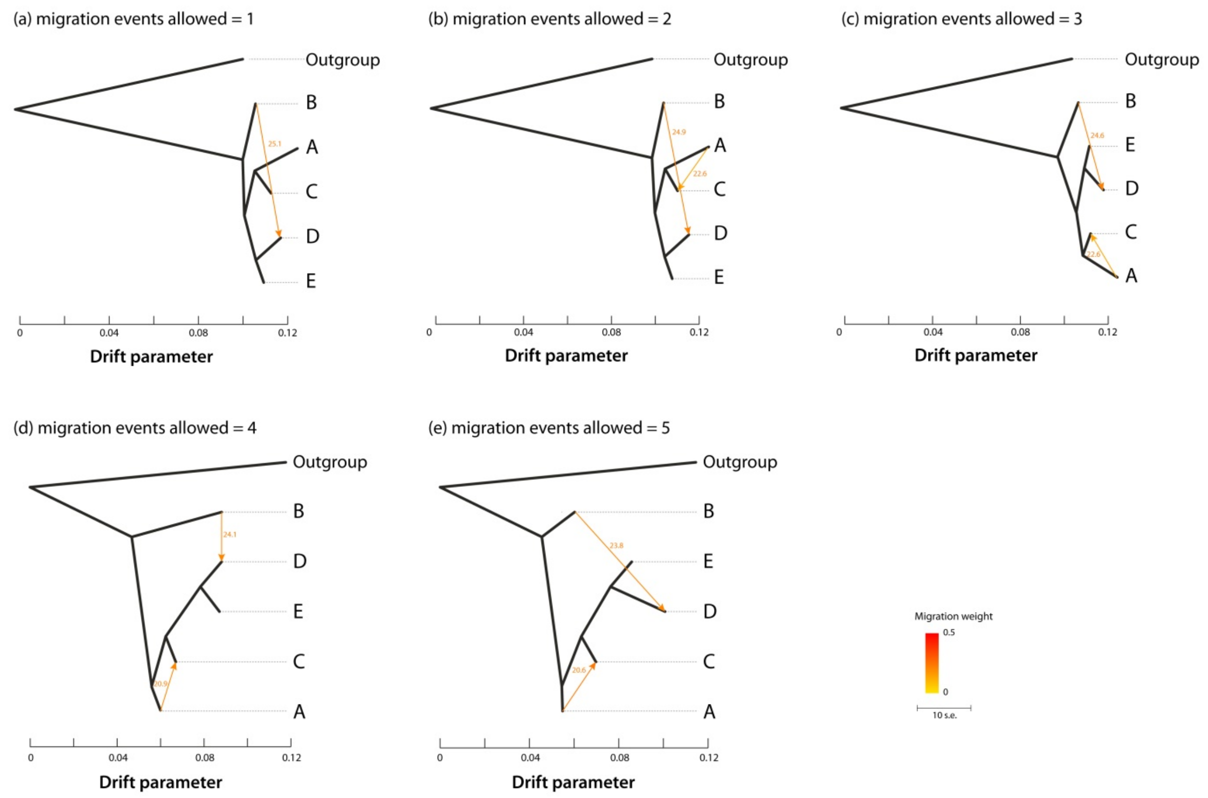

Fig. S7. Relatedness among *M. didyma* lineages recovered by *TreeMix*. The dataset included 25 specimens of *M. didyma* (five for each clade) and two specimens (*M. trivia* and *M. deione*) used as outgroup. The arrows (migration events) are coloured according to their weight. Only migration events that were statistically significant are shown. The migration weight represents the fraction of ancestry derived from the migration edge. The scale bar on the right shows the average standard error (s.e.) of the entries in the sample covariance matrix. Notable levels of gene flow are from B to D (Jackknife P=0.00012) and from A to C (P=0.00030).

Table S1. Specimens used in the study. COI, wsp and ftsZ sequences are publicly available in GenBank and in the dataset DS-DIDYMA (dx.doi.org/10.5883/DS-DIDYMA) from the Barcode of Life Data Systems (http://www.boldsystems.org/).

Sample ID	BOLD process ID (COI)	COI-5P Accession	ddRADseq data	ddRADseq lineage	COI lineage	*Wolbachia* infection	BOLD process ID (*Wolbachia*)	WSP Accession	WSP allele	ftsZ Accession	ftsZ allele	Taxon	Collection Date	Country	Exact Site	Lat	Lon	Elevation (m)
RVcoll.09-X232	EZSPC1374-10	HM901508	yes	C	L9	yes	LEWOL024-18	MN322898	wsp 64	MN322820	ftsZ 36	*M. didyma*	18-May-2009	France	Sault	44.071	5.356	870
RVcoll.12-Q005	WMB1763-13	MN138642	yes	C	L9	yes	LEWOL022-18	MN322893	wsp 64	MN322815	ftsZ 36	*M. didyma*	04-Aug-2012	France	Levant Island	43.186	6.333	355
RVcoll.12-P593	WMB3938-14	MN142285	yes	C	L9	yes	LEWOL023-18	MN322885	wsp 64	MN322807	ftsZ 36	*M. didyma*	2012-07-30	France	Cirque de Navacelles	43.884	3.5	630
RVcoll.14-H910	EULEP1979-15	MN143396	yes	C	L9	yes	LEWOL020-18	MN322896	wsp 10	MN322818	ftsZ 73	*M. didyma*	16-Jul-2014	France	Rouget	44.836	2.225	604
RVcoll10A449	EULEP5799-18	MN322873	yes	C	L9	yes	LEWOL025-18	MN322887	wsp 64	MN322809	ftsZ 36	*M. didyma*	2010-05-24	France	Pelissanne, A7 km240	43.60	5.17	85
RVcoll11I766	EULEP5807-18	MN322843	yes	C	L9	yes	LEWOL026-18	MN322895	wsp 64	MN322817	ftsZ 36	*M. didyma*	2011-07-31	France	Montauban-sur-l'Ouvèze	44.28	5.51	761
RVcoll11J199	EULEP5808-18	MN322882	yes	C	L9	yes	LEWOL027-18	MN322899	wsp 64	MN322821	ftsZ 36	*M. didyma*	2011-08-15	France	Col du Noyer	44.70	5.97	1539
RVcoll12O967	EULEP5821-18	MN322834	yes	C	L9	no						*M. didyma*	2012-08-12	France	Col de Menée	44.76	5.60	1441
RVcoll12P518	EULEP5822-18	MN322859	yes	C	L9	no						*M. didyma*	2012-07-30	France	La Calmette	43.91	4.29	93
RVcoll12Q067	EULEP5824-18	MN322880	yes	C	L9	yes	LEWOL028-18	MN322889	wsp 64	MN322811	ftsZ 36	*M. didyma*	2012-08-05	France	Collobrières	43.24	6.33	250
RVcoll12Q108	EULEP5825-18	MN322840	yes	C	L9	yes	LEWOL029-18	MN322886	wsp 64	MN322808	ftsZ 36	*M. didyma*	2012-08-06	France	Callas	43.57	6.57	260
RVcoll15H077	EULEP5859-18	MN322845	yes	C	L9	yes	LEWOL031-18	MN322888	wsp 64	MN322810	ftsZ 36	*M. didyma*	2015-07-05	Italy	Epinel	45.62	7.33	1530
RVcoll15L153	EULEP5862-18	MN322836	yes	C	L9	yes	LEWOL032-18	MN322892	wsp 64	MN322814	ftsZ 36	*M. didyma*	2015-07-15	Italy	Bormio	46.47	10.39	1300
RVcoll15M034	EULEP5864-18	MN322876	yes	C	L9	yes	LEWOL033-18	MN322894	wsp 64 (1 mutation)	MN322816	ftsZ 36	*M. didyma*	2015-07-10	Italy	La Palud	45.83	6.97	1480
RVcoll.08-P370	EZSPC551-09	GU669714	yes	E	L9	yes	LEWOL021-18	MN322897	wsp 64	MN322819	ftsZ 36	*M. didyma*	31-Jul-2008	Spain	Perves, Pallars Jussa	42.367	0.86	1071
RVcoll.08-J735	EZSPC1047-10	HM901250	yes	E	L9	no						*M. didyma*	14-Jun-2008	Spain	Piscifactoria de La Tosquilla, Titaguas	39.847	-1.145	586
RVcoll14V970	EULEP5856-18	MN322855	yes	E	L9	yes	LEWOL030-18	MN322890	wsp 64	MN322812	ftsZ 36	*M. didyma*	2015-06-03	Spain	Las Pedrosas	42.01	-0.85	
RVcoll.11-J146	EULEP646-15	MN144199	yes	C	L9	no						*M. didyma*	11-Aug-2011	Switzerland	Birguisch	46.327	7.971	1480
RVcoll.11-H582	WMB507-11	MN139924	yes	B	L2	no						*M. didyma - new lineage*	08-Jun-2011	Italy	San Martino alle Scale, Sicily	38.312	13.341	800
RVcoll.11-H748	WMB555-11	MN139967	yes	B	L2	no						*M. didyma - new lineage*	13-Jun-2011	Italy	San Teodoro, Sicily	37.85	14.71	1200
RVcoll.08-R519	WMB220-11	MN140467	yes	B	L2	no						*M. didyma - new lineage*	11-Apr-2011	Italy	Bosco de la Ficuzza, Sicily	37.838	13.429	923
RVcoll.11-I076	WMB627-11	MN142592	yes	B	L2	no						*M. didyma - new lineage*	18-Jun-2011	Italy	Randazzo, Sicily	37.943	15.08	1300
RVcoll.11-H995	WMB2916-14	MN143254	yes	B	L2	no						*M. didyma - new lineage*	17-Jun-2011	Italy	Piano Zucchi, Sicily	37.89	14.01	
RVcoll15K607	EULEP5861-18	MN322838	yes	C	L10	no						*M. didyma - new lineage*	2015-08-03	Italy	Mortola superiore	43.80	7.54	400
RVcoll.14-F516	EULEP1216-15	MN322831	yes	A	L4	no						*M. didyma liliputana*	02-Jul-2014	Greece	Pessani	41.047	26.081	143
RVcoll14F502	EULEP5828-18	MN322832	yes	A	L4	no						*M. didyma liliputana*	2014-07-02	Greece	Nipsa	40.96	26.07	243
RVcoll07F189	EULEP5795-18	MN322849	yes	A	L4	no						*M. didyma liliputana*	2007-08-09	Turkey	13 km N. of Saimbeily	38.07	36.15	1505
RVcoll07F194	EULEP5796-18	MN322881	yes	A	L4	no						*M. didyma liliputana*	2007-08-09	Turkey	13 km N. of Saimbeily	38.07	36.15	1505
RVcoll07F261	EULEP5797-18	MN322837	yes	A	L4	no						*M. didyma liliputana*	2007-08-14	Turkey	Erciyes Mountain	38.60	35.51	1870
RVcoll.08-J993	EZSPN680-09	GU676464	yes	E	L7	no						*M. didyma mauretanica*	17-Jul-2008	Spain	Casillas de Rojas, Gueejar Sierra	37.126	-3.441	1733
RVcoll.09-V584	EZSPC1226-10	HQ562783	yes	E	L7	no						*M. didyma mauretanica*	15-Jul-2009	Spain	Dornajo (Sierra Nevada)	37.122	-3.434	2060
RVcoll.11-D602	EZSPM604-12	KP870593	yes	E	L7	no						*M. didyma mauretanica*	13-May-2011	Spain	Canada Real de la Zubia, Monachil	37.087	-3.474	1684
RVcoll.14-F449	EULEP1184-15	MN322844	yes	A	L3	no						*M. didyma neera*	01-Jul-2014	Bulgaria	Rabovo, Arda Valley	41.611	25.654	186
RVcoll.14-F960	EULEP1370-15	MN322857	yes	A	L3	no						*M. didyma neera*	07-Jul-2014	Greece	Chelmos Mt., Xerocampos	38.009	22.199	1680
RVcoll.14-G115	EULEP1432-15	MN322878	yes	A	L3	no						*M. didyma neera*	09-Jul-2014	Greece	Katafigio - Profitis Ilias, Oros Taygetos	36.953	22.364	1645
RVcoll.14-G179	EULEP1453-15	MN322839	yes	A	L3	no						*M. didyma neera*	12-Jul-2014	Greece	Aposkepos	40.564	21.23	924
RVcoll.14-G632	EULEP1593-15	MN322853	yes	A	L3	no						*M. didyma neera*	16-Jul-2014	Greece	Romia	39.221	20.84	5
RVcoll.14-F524	EULEP531-15	MN322842	yes	A	L3	no						*M. didyma neera*	02-Jul-2014	Greece	Dadia	41.14	26.244	20
RVcoll11D714	EULEP5802-18	MN322848	yes	A	L3	no						*M. didyma neera*	2011-06-05	Kazakhstan	Kyzyl-Tas Mt.	48.28	85.43	786
RVcoll11G386	EULEP5803-18	MN322868	yes	A	L3	no						*M. didyma neera*	2011-05-30	Kazakhstan	Chirkain	49.38	84.26	864
RVcoll11G595	EULEP5804-18	MN322865	yes	A	L3	no						*M. didyma neera*	2011-06-01	Kazakhstan	Kalgutinsky Pass	48.48	84.11	718
RVcoll11G663	EULEP5805-18	MN322833	yes	A	L3	no						*M. didyma neera*	2011-06-03	Kazakhstan	Mramorugi Pass	48.45	85.88	855
RVcoll.08-M265	EZRMN113-08	HQ004789	yes	A	L3	no						*M. didyma neera*	27-May-2008	Romania	4 Km W of Drobeta Turnu-Severin	44.638	22.589	75
RV-06-M973	EZROM417-08	HQ004793	yes	A (not included in Fig. 1)	L3	no						*M. didyma neera*	20-Jul-2006	Romania	Izvorul Muresului	46.63	25.695	870
RVcoll14E926	EULEP5827-18	MN322870	yes	A	L3	no						*M. didyma neera*	2014-06-26	Serbia	Povlen Mt., near Pasna Ravan	44.16	19.69	1092
RVcoll.14-N358	EULEP2198-15	MN322851	yes	A	L3	no						*M. didyma neera*	12-Jul-2014	Ukraine	Mali Dmytrovychi	50.22	30.52	160
RVcoll.14-V093	EULEP2390-15	MN322871	yes	A	L3	no						*M. didyma neera*	05-Jul-2014	Ukraine	Kitsevka, Pechenegi distr.	49.85	36.833	
RVcoll.14-V402	EULEP2528-15	MN322869	yes	A	L3	no						*M. didyma neera*	25-Jun-2014	Ukraine	Sudak dist., Mandzil-Kaja Mt.	44.91	34.99	
RVcoll.12-Q313	WMB3986-14	MN138697	yes	C	L5	no						*M. didyma neera 2*		France	Bezaudun-les-Alpes	43.801	7.094	885
RVcoll.12-O756	EULEP748-15	MN142497	yes	C	L5	no						*M. didyma neera 2*	09-Aug-2012	France	Col de la Lombarde	44.198	7.152	2280
RVcoll.11-I689	WMB730-12	MN144593	yes	C	L5	no						*M. didyma neera 2*	30-Jul-2011	France	Le Poeet-en-Percip	44.255	5.398	1096
RVcoll10A578	EULEP5800-18	MN322864	yes	C	L5	no						*M. didyma neera 2*	2010-05-25	France	Digne les Bains	44.08	6.19	660
RVcoll12O695	EULEP5819-18	MN322872	yes	C	L5	no						*M. didyma neera 2*	2012-08-09	France	Isola	44.21	7.10	1590
RVcoll12P738	EULEP5823-18	MN322866	yes	C	L5	no						*M. didyma neera 2*	2012-08-01	France	Plan-d´Aups-Sainte-Baume	43.32	5.70	685
RVcoll.11-J622	WMB739-12	MH419035	yes	C	L5	no						*M. didyma neera 2*	15-Jul-2011	Italy	Orbetello	42.43	11.37	1
RVcoll.12-O043	WMB2965-14	MH419443	yes	C	L5	no						*M. didyma neera 2*	10-Jun-2012	Italy	Argentario	42.428	11.159	51
RVcoll.12-R360	WMB2184-13	MH419894	yes	C	L5	no						*M. didyma neera 2*	25-Jul-2012	Italy	San Piero, Elba Island	42.75	10.2	300
RVcoll.13-S727	WMB4166-14	MN142615	yes	C	L5	no						*M. didyma neera 2*		Italy	Guarcino	41.82	13.32	1200
RVcoll.11-I119	WMB720-12	MN144597	yes	C	L5	no						*M. didyma neera 2*	19-Jun-2011	Italy	San Luca	38.201	15.98	1500
RVcoll.14-N085	OXB371-15	MN144656	yes	C	L5	no						*M. didyma neera 2*	27-Jun-2014	Italy	Galzignano	45.31	11.696	310

Sample ID	BOLD process ID (COI)	COI-5P Accession	ddRADseq data	ddRADseq lineage	COI lineage	*Wolbachia* infection	BOLD process ID (*Wolbachia*)	WSP Accession	WSP allele	ftsZ Accession	ftsZ allele	Taxon	Collection Date	Country	Exact Site	Lat	Lon	Elevation (m)
RVcoll.12-O903	WMB3890-14	MN144708	yes	C	L5	no						*M. didyma neera 2*		Italy	Bersezio	44.383	6.966	1630
RVcoll.12-Q922	WMB2363-13	MN144736	yes	C	L5	no						*M. didyma neera 2*	31-Jul-2012	Italy	Aspromonte, Contrada Sclano, S	38.067	15.817	1175
13-U302	OXB741-15	MN145183	yes	C	L5	no						*M. didyma neera 2*	15-Jul-2013	Italy	Val d`Oten	46.482	12.315	1321
RVcoll14A356	EULEP5826-18	MN322850	yes	C	L5	no						*M. didyma neera 2*	2013-07-26	Italy	Foce di Montemonaco	42.87	13.27	1000
RVcoll14W669	EULEP5857-18	MN322861	yes	C	L5	no						*M. didyma neera 2*	2014-07-22	Italy	Nago-torbole	45.88	10.93	170
RVcoll14W858	EULEP5858-18	MN322883	yes	C	L5	no						*M. didyma neera 2*	2014-08-05	Italy	Magliolo	44.20	8.25	350
RVcoll15L572	EULEP5863-18	MN322884	yes	C	L5	no						*M. didyma neera 2*	2015-05-11	Italy	Fornovo di Taro	44.69	10.07	
RVcoll15M518	EULEP5865-18	MN322867	yes	C	L5	no						*M. didyma neera 2*	2015-07-09	Italy	Trecate, polo industriale San Martina	45.45	8.79	130
RVcoll.10-B979	WMB3522-14	MN143228	yes	C	L1	no						*M. didyma occidentalis*	12-Aug-2010	France	Vars	44.631	6.677	1570
RVcoll.11-I690	WMB731-12	MN143860	yes	C	L1	no						*M. didyma occidentalis*	30-Jul-2011	France	Le Poeet-en-Percip	44.255	5.398	1096
RVcoll10B993	EULEP5801-18	MN322856	yes	C	L1	no						*M. didyma occidentalis*	2010-08-13	France	Road to Col de l'Isoard	44.81	6.74	2000
RVcoll11I691	EULEP5806-18	MN322852	yes	C	L1	no						*M. didyma occidentalis*	2011-07-30	France	Le Pöet-en-Percip	44.26	5.40	1096
RVcoll12O852	EULEP5820-18	MN322846	yes	C	L1	no						*M. didyma occidentalis*	2012-08-11	France	Larche (camping Les Marmottes)	44.45	6.85	1680
RVcoll15J468	EULEP5860-18	MN322862	yes	C	L1	no						*M. didyma occidentalis*	2015-07-25	France	Prorel (around church Notre-Dame des Neiges)	44.90	6.59	2190-2300
RVcoll.08-P048	EZSPC552-09	GU669715	yes	E	L1	no						*M. didyma occidentalis*	19-Aug-2008	Spain	La Valira (near Urus), Cerdanya	42.35	1.85	1300
RVcoll.08-J709	EZSPC1046-10	HM901249	yes	E	L1	no						*M. didyma occidentalis*	01-May-2008	Spain	Alrededores de Almedijar, Almedijar	39.875	-0.417	
RVcoll.08-J737	EZSPC1048-10	HM901251	yes	E	L1	no						*M. didyma occidentalis*	14-Jun-2008	Spain	Casa de Camineros, Tuejar	39.822	-1.13	581
RVcoll.08-L915.1	EZSPC1092-10	HM901293	yes	E	L1	no						*M. didyma occidentalis*	14-Jul-2008	Spain	Escamilla	40.539	-2.62	1040
RVcoll.08-L951	EZSPC1099-10	HM901300	yes	E	L1	no						*M. didyma occidentalis*	21-Jul-2008	Spain	Puerto de Pozazal	42.914	-4.111	1003
RVcoll08J742	EULEP5798-18	MN322841	yes	E	L1	no						*M. didyma occidentalis*	2008-06-14	Spain	Casa de Camineros, Tuéjar	39.82	-1.13	581
RVcoll15Q052	EULEP5893-18	MN322874	yes	D	L6	no						*M. didyma protaeoccidentis*	2015-06-04	Algeria	Cedraie et chênaie verte de Tikjda-	36.45	4.11	1479
RVcoll.12-R298	WMB2145-13	MN322858	yes	D	L6	no						*M. didyma protaeoccidentis*	15-Jun-2012	Morocco	Oukaimeden et env., N djebel Toubkal	31.09	-7.915	2750
RVcoll.06-A056	WMB3105-14	MN322879	yes	D	L6	no						*M. didyma protaeoccidentis*	24-May-2006	Morocco	Ouled Ayad, Azilal, Middle Atlas	32.176	-6.792	700
RVcoll.11-F879	WMB3625-14	MN322854	yes	D	L6	no						*M. didyma protaeoccidentis*	01-Jul-2011	Morocco	Col du Zad, Middle Atlas	32.993	-5.075	2255
RVcoll.12-L827	WMB3732-14	MN322875	yes	D	L6	no						*M. didyma protaeoccidentis*	17-May-2012	Morocco	Talassemtane, Chefchaouen	35.075	-5.168	1137
RVcoll.11-F172	WMB387-11	MN322835	yes	D	L6	no						*M. didyma protaeoccidentis*	25-Jun-2011	Morocco	Jbel Lakraa	35.119	-5.194	882
RVcoll.13-S473	WMB4096-14	MN322860	yes	D	L6	no						*M. didyma protaeoccidentis*		Morocco	W Monts de Beni-Snassen (Taforalt Zegzel), S Berkane	34.83	-2.38	
RVcoll.06-G603	WMB774-13	MN322863	yes	D	L6	no						*M. didyma protaeoccidentis*	30-May-2006	Morocco	Ijoukak, High Atlas	31.087	-8.131	1100
RVcoll.12-N510	WMB1407-13	MN322847	yes	D	L6	yes	LEWOL019-18	MN322891	**wsp new**	MN322813	ftsZ 7	*M. didyma protaeoccidentis*	01-Jun-2012	Tunisia	Ayn Darahim, Jendouba	36.764	8.666	660
RVcoll.12-Q342	WMB1804-13	MN144241	yes	C	L8	no						*M. didyma turkestanica 2*	08-Aug-2012	France	Valdeblore, Alpes-Maritimes	44.069	7.208	1300
RVcoll.10-C562	WMB2724-13	MN138770	yes	C	L8	no						*M. didyma turkestanica 2*	10-Jun-2010	Italy	Argentario	42.428	11.159	51
12-R197	BIBSA956-15	MN143451	yes	C	L8	no						*M. didyma turkestanica 2*	05-Aug-2012	Italy	Aurunci, Monte S. Angelo, Italian coast hills	41.3	13.617	743
RVcoll.11-J247	WMB3692-14	MN138695	yes	E	L8	no						*M. didyma turkestanica 2*	26-Aug-2011	Spain	Mollet de Peralada, Alt Emporda	42.363	2.998	56
RVcoll15A753	EULEP5919-18	MN322877	yes	outgrou		no						*M. deione*	2015-07-06	Portugal	Trancoso	40.764	-7.412	794
RVcoll14I563	WMB5016-14	MN144039	yes	outgrou		no						*M. trivia*	2011-06-16	Italy	Val Rosandra	45.61	13.87	
M8		KY086102										*M. casta*		Iran				
M9		KY086103										*M. casta*		Iran				
NIBGE BUT-00254	MABUT254-11	KC158426										*M. chitralensis*	20-Jul-2010	Pakistan	Chitral	35.8333	71.7667	1514
NIBGE BUT-00253	MABUT253-11	KC158427										*M. chitralensis*	20-Jul-2010	Pakistan	Chitral	35.8333	71.7667	1514
	BPAL3124-15	KY086157										*M. deserticola*		Israel	Jerusalem			
	BPALB134-16	KY086186										*M. deserticola*		Israel	Hermon			
	BPALB150-16	KY086193										*M. deserticola*		Israel	Hermon			
		AF187759										*M. deserticola*		Lebanon	Mohafazat Zahle			
	BPAL2353-14	KY086107										*M. deserticola*		Syria	Damaskus			
	BPAL2354-14	KY086108										*M. deserticola*		Syria	Damaskus			
CCDB-17966 H05	BPAL2559-14	KT874721										*M. didyma*	10-Aug-1990	Austria	Tirol			
CCDB-17966 C01	BPAL2495-14	KT874733										*M. didyma*	10-Aug-1990	Austria	Tirol			
CCDB-17966 H04	BPAL2558-14	KT874743										*M. didyma*	10-Aug-1990	Austria	Tirol			
AF187763	GBLN0057-06	AF187763										*M. didyma*		France	Montpellier, Languedoc			
FJ462252	GBLN1856-09	FJ462252										*M. didyma*		France	Aude			
BC ZSM Lep 21547	GWORK312-09	JF415717										*M. didyma*	18-Jun-1992	Germany	Moernsheim, Steinbruch 2 km w	48.8619	10.9967	430
RVcoll.08-M960	EZSPN1026-09	GU676138										*M. didyma*	06-Jul-2008	Spain	Saganta	42.012	0.478	767
RVcoll.08-P929	EZSPC1149-10	HM901349										*M. didyma*	01-Aug-2009	Spain	Yatova, Sierra Martes	39.339	-0.976	900
RVcoll.06-G485	EZSPC974-10	HM901809										*M. didyma*	08-May-2006	Spain	Barranco de Valcuerna, Candasnos	41.465	0.02	210
RVcoll.06-G491	EZSPC975-10	HM901810										*M. didyma*	08-May-2006	Spain	Barranco de Valcuerna, Candasnos	41.465	0.02	210
Rvcoll. 090111XZ00	EZSPM797-12	KP870744										*M. didyma*	23-Jul-2002	Spain	Balneario de Panticosa	42.76	-0.237	1642
RVcoll.08-J030	EZSPN539-09	GU676943										*M. didyma*	22-Apr-2008	Spain	Teresa de Cofrentes, arroyo Cangrejos, Cofrentes	39.11	-1.062	
CCDB-17966 C07	BPAL2501-14	KT874748										*M. didyma liliputana*	20-Jun-1999	Armenia	Tuva, 30 km N Kyzyl			
	LOWAB015-07	KY118818										*M. didyma liliputana*		Armenia				
	LOWAB016-07	KY118819										*M. didyma liliputana*		Armenia				
	LOWAB063-07	KY118820										*M. didyma liliputana*		Armenia				

Sample ID	BOLD process ID (COI)	COI-5P Accession	ddRADseq data	ddRADseq lineage	COI lineage	*Wolbachia* infection	BOLD process ID (*Wolbachia*)	WSP Accession	WSP allele	ftsZ Accession	ftsZ allele	Taxon	Collection Date	Country	Exact Site	Lat	Lon	Elevation (m)
CCDB-17968 E10	BPAL2718-14	KT874744										*M. didyma liliputana*	03-Jul-2014	Israel	Hermon			
	BPALB135-16	KY086187										*M. didyma liliputana*		Israel	Hermon			
	BPALB136-16	KY086188										*M. didyma liliputana*		Israel	Hermon			
	BPALB137-16	KY086189										*M. didyma liliputana*		Israel	Hermon			
	BPALB139-16	KY086190										*M. didyma liliputana*		Israel	Hermon			
	BPALB142-16	KY086191										*M. didyma liliputana*		Israel	Hermon			
	BPALB143-16	KY086192										*M. didyma liliputana*		Israel	Hermon			
CCDB-17951 B04	BPAL2296-14	KT874738										*M. didyma liliputana*	02-May-1995	Syria	Damaskus, vic. Dimas			
	BPAL3293-16	KY086166										*M. didyma liliputana*		Turkey	Torul			
	BPAL3294-16	KY086167										*M. didyma liliputana*		Turkey	Torul			
RVcoll.09-V572	EZSPC1224-10	HQ562781										*M. didyma mauretanica*	15-Jul-2009	Spain	Dornajo (Sierra Nevada)	37.122	-3.434	2060
RVcoll.09-V583	EZSPC1225-10	HQ562782										*M. didyma mauretanica*	15-Jul-2009	Spain	Dornajo (Sierra Nevada)	37.122	-3.434	2060
RVcoll.11-D609	EZSPM605-12	KP870659										*M. didyma mauretanica*	13-May-2011	Spain	Canada Real de la Zubia, Monachil	37.087	-3.474	1684
KT792908	GBMIN85907-17	KT792908										*M. didyma neera*	30-Jun-2017	Bulgaria	Selva del Turchese	40.1287	18.4735	
	BPAL2969-15	KY086126										*M. didyma neera*		Bulgaria	Emen			
	BPAL3087-15	KY086154										*M. didyma neera*		Bulgaria	Hvoyna			
M27		KY086212										*M. didyma neera*		Bulgaria	Hvoyna			
	BPAL3086-15	KY086153										*M. didyma neera*		Greece	Pades			
	BPAL3164-16	KY086158										*M. didyma neera*		Greece	Timfristos			
	BPAL3165-16	KY086159										*M. didyma neera*		Greece	Kalavrita			
	BPAL3166-16	KY086160										*M. didyma neera*		Greece	Kalavrita			
	BPAL3167-16	KY086161										*M. didyma neera*		Greece	Kalavrita			
2005-LOWA-824	LOWA824-06	FJ663786										*M. didyma neera*	07-Jun-1998	Kazakhstan		49.533	83.817	450
2005-LOWA-823	LOWA823-06	FJ663787										*M. didyma neera*	07-Jun-1998	Kazakhstan		49.533	83.817	450
2005-LOWA-822	LOWA822-06	FJ663788										*M. didyma neera*	07-Jun-1998	Kazakhstan		49.533	83.817	450
CCDB-17966 C05	BPAL2499-14	KT874709										*M. didyma neera*	07-Jul-1996	Kazakhstan	Aktiobe			
	BPAL2767-15	KY086112										*M. didyma neera*		Kazakhstan	Karabiryuk			
	BPAL2946-15	KY086121										*M. didyma neera*		Kazakhstan	Altai, Chirkain			
	BPAL2947-15	KY086122										*M. didyma neera*		Kazakhstan	Altai, Chirkain			
	BPAL2997-15	KY086133										*M. didyma neera*		Kazakhstan	Karabiryuk			
	BPAL3478-16	KY086172										*M. didyma neera*		Kazakhstan	Aktobe			
	BPAL3479-16	KY086173										*M. didyma neera*		Kazakhstan	Aktobe			
	BPAL3480-16	KY086174										*M. didyma neera*		Kazakhstan	Aktobe			
	BPAL3482-16	KY086175										*M. didyma neera*		Kazakhstan	Aktobe			
	BPAL3488-16	KY086176										*M. didyma neera*		Kazakhstan	Turgusun			
RVcoll.08-M300	EZRMN114-08	HQ004790										*M. didyma neera*	31-May-2008	Romania	Suatu, Cluj	46.796	23.96	400
RV-06-M995	EZROM418-08	HQ004791										*M. didyma neera*	18-Jul-2007	Romania	Apuseni Mts., Rimetea	46.452	23.582	750
RV-06-M962	EZROM416-08	HQ004792										*M. didyma neera*	31-Jul-2006	Romania	Racos, Cluj	46.031	25.366	460
RVcoll.10-C421	EULEP143-14	KP870704										*M. didyma neera*	24-Jul-2010	Romania	Prislop village, Maramures	47.41	23.55	
RVcoll.11-J885	EULEP187-14	KP870749										*M. didyma neera*	06-Jul-2011	Romania	Padurea Hoia, Cluj	46.768	23.516	
FJ462249	GBLN1859-09	FJ462249										*M. didyma neera*		Russia	Udmurtia Kilmez'			
KT792898	GBMIN85911-17	KT792898										*M. didyma neera*		Russia	Samara oblast	53.48	50.16	
188031		KT792899										*M. didyma neera*	29 June 2014	Russia	Tugustemir, Nakas			
KT792900	GBMIN85908-17	KT792900										*M. didyma neera*		Russia	Bashkiria, Zilair	41.87	24.67	
KT792901	GBMIN85912-17	KT792901										*M. didyma neera*		Russia	Samara	53.48	50.16	
226015		KT792902										*M. didyma neera*	27 June 2014	Russia	Volzhskiy	53°4'87.7	50°0'86.9	
KT792903	GBMIN85910-17	KT792903										*M. didyma neera*		Russia	Samara	53.48	50.16	
KT792905	GBMIN85913-17	KT792905										*M. didyma neera*		Russia	Samara	53.48	50.16	
CCDB-03033 H03	BPAL1702-12	KT874706										*M. didyma neera*	12-Aug-2011	Russia	North Ossetia, pass Archonsky. N. E.			2900
CCDB-03033 G03	BPAL1690-12	KT874713										*M. didyma neera*	27-May-2010	Russia	Daghestan, Makhachkala, Talgi. N. E.			300
CCDB-03033 H08	BPAL1707-12	KT874716										*M. didyma neera*	04-Jun-2009	Russia	Daghestan, Makhachkala, Talgi. N. E.			300
CCDB-17966 G12	BPAL2554-14	KT874720										*M. didyma neera*	05-Jul-1998	Russia	Kaluga			
CCDB-17966 H01	BPAL2555-14	KT874730										*M. didyma neera*	05-Jul-2001	Russia	Voronezh			
	BPAL3023-15	KY086141										*M. didyma neera*		Russia	Orenburg distr, Mednogorsk			
	BPAL3035-15	KY086142										*M. didyma neera*		Russia	Orenburg distr, Mednogorsk			
	BPAL3036-15	KY086143										*M. didyma neera*		Russia	Orenburg distr, Luch Truda			
	BPAL3044-15	KY086144										*M. didyma neera*		Russia	Samarskaya obl, Bachilova polyana			
	BPAL3048-15	KY086145										*M. didyma neera*		Russia	Samarskaya obl, Bachilova polyana			
	BPALB186-16	KY086194										*M. didyma neera*		Russia	Khakassia			
	BPALB187-16	KY086195										*M. didyma neera*		Russia	Khakassia			
	BPALB190-16	KY086196										*M. didyma neera*		Russia	Khakassia			
	BPALB189-16	KY086197										*M. didyma neera*		Russia	Khakassia			
	BPAL3089-15	KY086155										*M. didyma neera*		Slovenia	Jagnjenica			
	BPAL3090-15	KY086156										*M. didyma neera*		Slovenia	Jagnjenica			
FJ462251	GBLN1857-09	FJ462251										*M. didyma occidentalis*		Spain				

Sample ID	BOLD process ID (COI)	COI-5P Accession	ddRADseq data	ddRADseq lineage	COI lineage	*Wolbachia* infection	BOLD process ID (*Wolbachia*)	WSP Accession	WSP allele	ftsZ Accession	ftsZ allele	Taxon	Collection Date	Country	Exact Site	Lat	Lon	Elevation (m)
RVcoll.06-H891	EZSPC549-09	GU669712										*M. didyma occidentalis*	11-May-2006	Spain	El Polell, P.N. del Montseny	41.739	2.392	780
RVcoll.08-L440	EZSPC550-09	GU669713										*M. didyma occidentalis*	21-May-2008	Spain	Balsareny, Bages	41.874	1.879	363
RVcoll.08-R068	EZSPM355-09	GU675881										*M. didyma occidentalis*	05-Jul-2009	Spain	La Cerollera	40.841	-0.047	781
RVcoll.08-L832	EZSPN908-09	GU676247										*M. didyma occidentalis*	10-Jul-2008	Spain	Oteruelo del Valle	40.891	-3.849	1152
RVcoll.08-L812	EZSPN900-09	GU676256										*M. didyma occidentalis*	04-Jul-2008	Spain	San Andres	42.913	-4.092	972
RVcoll.08-L234	EZSPN757-09	GU676395										*M. didyma occidentalis*	08-Aug-2008	Spain	Ruta de la Sima, El Vallecillo	40.257	-1.607	1531
RVcoll.08-H692	EZSPC1017-10	HM901221										*M. didyma occidentalis*	03-Aug-2008	Spain	Huelamo, Rio Jucar	40.316	-1.805	1237
RVcoll.08-L204	EZSPC1067-10	HM901270										*M. didyma occidentalis*	27-Jul-2008	Spain	La Vega, Vilafranca	40.429	-0.268	1124
RVcoll.08-L728	EZSPC1082-10	HM901283										*M. didyma occidentalis*	18-Jun-2008	Spain	Arganda del Rey	40.289	3.426	689
RVcoll.08-L914	EZSPC1091-10	HM901292										*M. didyma occidentalis*	14-Jul-2008	Spain	Escamilla	40.539	-2.62	1040
RVcoll.07-F075	EZSPC990-10	HM901825										*M. didyma occidentalis*	05-Aug-2007	Spain	10 km S. of Morella	40.539	-0.146	900
FJ462253	GBLN1855-09	FJ462253										*M. didyma protaeoccidentis*		Morocco	Atlas, 3 km S Col du Zad			
CCDB-17951 B05	BPAL2297-14	KT874712										*M. didyma protaeoccidentis*	26-May-1980	Morocco	M. Atlas, Col du Zad			
RPVL-00098	BPAL098-10	HM404715										*M. didyma turkestanica*		China	Urumchi Khami Nanshangou			1850
RPVL-00099	BPAL099-10	HM404716										*M. didyma turkestanica*		China	Urumchi Khami Nanshangou			1850
RPVL-00102	BPAL102-10	HM404718										*M. didyma turkestanica*		China	Urumchi Khami Karlyktag			2300
CCDB-17951 A10	BPAL2290-14	KT874698										*M. didyma turkestanica*	17-Jun-2007	China	Urumchi			
CCDB-17951 A11	BPAL2291-14	KT874725										*M. didyma turkestanica*	17-Jun-2007	China	Urumchi			
CCDB-17966 C02	BPAL2496-14	KT874695										*M. didyma turkestanica*	12-Jun-1998	Kazakhstan	Altai, Tchernyi Irtysh			
CCDB-17966 C06	BPAL2500-14	KT874703										*M. didyma turkestanica*	07-Jul-1996	Kazakhstan				
CCDB-17966 C09	BPAL2503-14	KT874707										*M. didyma turkestanica*	09-Jun-2000	Kazakhstan	Alma-Ata region			
CCDB-17966 C08	BPAL2502-14	KT874718										*M. didyma turkestanica*	09-Jun-2000	Kazakhstan	Alma-Ata region			
CCDB-17966 C04	BPAL2498-14	KT874735										*M. didyma turkestanica*	12-Jun-1998	Kazakhstan	Altai, Tchernyi Irtysh			
CCDB-17966 C03	BPAL2497-14	KT874737										*M. didyma turkestanica*	12-Jun-1998	Kazakhstan	Altai, Tchernyi Irtysh			
	BPAL2556-14	KY086110										*M. didyma turkestanica*		Kazakhstan	Karzhantau			
	BPAL2766-15	KY086111										*M. didyma turkestanica*		Kazakhstan	Karabiryuk			
	BPAL2768-15	KY086113										*M. didyma turkestanica*		Kazakhstan	Karabiryuk			
	BPAL2769-15	KY086114										*M. didyma turkestanica*		Kazakhstan	Karabiryuk			
	BPAL2770-15	KY086115										*M. didyma turkestanica*		Kazakhstan	Saikan			
	BPAL2771-15	KY086116										*M. didyma turkestanica*		Kazakhstan	Saikan			
	BPAL2772-15	KY086117										*M. didyma turkestanica*		Kazakhstan	Saikan			
	BPAL2991-15	KY086130										*M. didyma turkestanica*		Kazakhstan	Karabiryuk			
	BPAL2992-15	KY086131										*M. didyma turkestanica*		Kazakhstan	Karabiryuk			
	BPAL2995-15	KY086132										*M. didyma turkestanica*		Kazakhstan	Karabiryuk			
	BPAL2998-15	KY086134										*M. didyma turkestanica*		Kazakhstan	Karabiryuk			
	BPAL2999-15	KY086135										*M. didyma turkestanica*		Kazakhstan	Karabiryuk			
	BPAL3496-16	KY086179										*M. didyma turkestanica*		Kazakhstan	Kaindy			
	BPAL3512-16	KY086183										*M. didyma turkestanica*		Kazakhstan	Tentek Ucharal			
	BPAL3513-16	KY086184										*M. didyma turkestanica*		Kazakhstan	Karzhan			
	BPAL3514-16	KY086185										*M. didyma turkestanica*		Kazakhstan	Karzhan			
FJ462250	GBLN1858-09	FJ462250										*M. didyma turkestanica*		Kyrgyzstan	Suusamyr Mts Kyzyl-Oi			
FJ462269	GBLN1840-09	FJ462269										*M. didyma turkestanica*		Kyrgyzstan	N. Chatkal Chamach			
CCDB-17966 C10	BPAL2504-14	KT874710										*M. didyma turkestanica*	20-Jul-1992	Kyrgyzstan	Kadzhi-Sai			
CCDB-17966 C11	BPAL2505-14	KT874742										*M. didyma turkestanica*	20-Jul-1992	Kyrgyzstan	Kadzhi-Sai			
CCDB-17966 H03	BPAL2557-14	KT874746										*M. didyma turkestanica*	30-Jun-2001	Kyrgyzstan	Talassky Mts, Kara-Bura Pass			
	BPAL3503-16	KY086180										*M. didyma turkestanica*		Kyrgyzstan	Talas Mts Kara-Bura			
	BPAL3504-16	KY086181										*M. didyma turkestanica*		Kyrgyzstan	Talas Mts Kara-Bura			
	BPAL3505-16	KY086182										*M. didyma turkestanica*		Kyrgyzstan	Talas Mts Kara-Bura			
KT792904	GBMIN85909-17	KT792904										*M. didyma turkestanica*		Russia	Samara	52.25	57.52	
	BPAL3168-16	KY086162										*M. didyma turkestanica*		Russia	Astrakhan oblast			
	BPAL3169-16	KY086163										*M. didyma turkestanica*		Russia	Astrakhan oblast			
	BPAL3170-16	KY086164										*M. didyma turkestanica*		Russia	Astrakhan oblast			
	BPAL3173-16	KY086165										*M. didyma turkestanica*		Russia	Astrakhan oblast			
CCDB-17966 D04	BPAL2510-14	KT874694										*M. didyma turkestanica*	04-Jun-1994	Tajikistan	Kuraminski Mts Altyn Topkan			
CCDB-17966 D05	BPAL2511-14	KT874715										*M. didyma turkestanica*	04-Jun-1994	Tajikistan	Kuraminski Mts Altyn Topkan			
CCDB-17966 C12	BPAL2506-14	KT874726										*M. didyma turkestanica*	13-Jun-1994	Uzbekistan	Nuratau, Sarmitan			
CCDB-17966 D01	BPAL2507-14	KT874745										*M. didyma turkestanica*	13-Jun-1994	Uzbekistan	Nuratau Sarmitan			
	BPAL2507-14	KY086109										*M. didyma turkestanica*		Uzbekistan	Nuratau, Sarmitan			
CCDB-17966 G05	BPAL2547-14	KT874727										*M. didymoides*	01-Jul-1992	China	Beijing Municipality			
CCDB-17966 G04	BPAL2546-14	KT874750										*M. didymoides*	24-Jul-2002	Mongolia	Selenge Aimak			
AF187762	GBLN0056-06	AF187762										*M. didymoides*		Russia	Buryatia, Dodo-Enchor			
	BPAL3375-16	KY086168										*M. didymoides*		Russia	Buryatia			
	BPAL3493-16	KY086178										*M. didymoides*		Russia	Buryatia			
	BPAL1697-12	KY086104										*M. gina*		Azerbaijan	Azerbaijan, Shamkir			
		FJ462258										*M. gina*		Iran	Dascht-e-Arjan			

Sample ID	BOLD process ID (COI)	COI-5P Accession	ddRADseq data	ddRADseq lineage	COI lineage	*Wolbachia* infection	BOLD process ID (*Wolbachia*)	WSP Accession	WSP allele	ftsZ Accession	ftsZ allele	Taxon	Collection Date	Country	Exact Site	Lat	Lon	Elevation (m)
	BPAL2350-14	KY086106										*M. gina*		Iran	30 km S Marivan			
	BPAL2783-15	KY086118										*M. gina*		Iran	Kuh-e-Tamandar			
	BPAL2784-15	KY086119										*M. gina*		Iran	Kuh-e-Tamandar			
	BPAL2786-15	KY086120										*M. gina*		Iran	Kuh-e-Tamandar			
	BPAL2951-15	KY086123										*M. gina*		Iran	Gilan			
	BPAL2952-15	KY086124										*M. gina*		Iran	Gilan			
M1		KY086198										*M. gina*		Iran	Zedasht			
M10		KY086199										*M. gina*		Iran	E of Senandaj			
M11		KY086200										*M. gina*		Iran	E of Senandaj			
M12		KY086201										*M. gina*		Iran	E of Senandaj			
M14 (Q156)		KY086202										*M. gina*		Iran	27 E of Mahabad			
M15 (Q157)		KY086203										*M. gina*		Iran	27 E of Mahabad			
M16 (Q158)		KY086204										*M. gina*		Iran	27 E of Mahabad			
M17		KY086205										*M. gina*		Iran	27 E of Mahabad			
M2		KY086206										*M. gina*		Iran	Zedasht			
M20		KY086207										*M. gina*		Iran	23 E of Mahabad			
M21		KY086208										*M. gina*		Iran	W of Mahabad			
M22		KY086209										*M. gina*		Iran	Seir			
M23		KY086210										*M. gina*		Iran	Seir			
M25		KY086211										*M. gina*		Iran	Iran, 2nd pass			
M3		KY086213										*M. gina*		Iran	Zedasht			
M4		KY086214										*M. gina*		Iran	Zedasht			
M5		KY086215										*M. gina*		Iran	Soutekesh			
M7		KY086216										*M. gina*		Iran	Saravand			
	BPAL3068-15	KY086146										*M. gina*		Iran	Khansar			
	BPAL3069-15	KY086147										*M. gina*		Iran	Khansar			
	BPAL3078-15	KY086148										*M. gina*		Iran	Fereydun Shahr			
	BPAL3079-15	KY086149										*M. gina*		Iran	Saqgez-Baneh			
	BPAL3081-15	KY086150										*M. gina*		Iran	N Chenareh			
	BPAL3082-15	KY086151										*M. gina*		Iran	E of Schndag			
	BPAL3083-15	KY086152										*M. gina*		Iran	E of Schndag			
CCDB-17966 F10	BPAL2540-14	KT874702										*M. interrupta*	23-Jul-1999	Armenia	Megri			
CCDB-17966 F11	BPAL2541-14	KT874740										*M. interrupta*	22-Jul-1999	Armenia	Kadjaran vs. Kotayk`			
CCDB-17966 F09	BPAL2539-14	KT874741										*M. interrupta*	23-Jul-1999	Armenia	Megri			
CCDB-03033 H07	BPAL1706-12	KT874734										*M. interrupta*	07-Aug-2010	Daghestan	Daghestan, Kurach. N. E.			2100
	BPAL2968-15	KY086125										*M. interrupta*		Georgia	Akhaldoba			
	BPAL2971-15	KY086127										*M. interrupta*		Georgia	Sagrdze			
	BPAL2972-15	KY086128										*M. interrupta*		Georgia	Adjara, Chanchkhalo			
	BPAL2973-15	KY086129										*M. interrupta*		Georgia	Adjara, Chanchkhalo			
	BPAL3001-15	KY086136										*M. interrupta*		Georgia	Akhaltsikhe			
	BPAL3002-15	KY086137										*M. interrupta*		Georgia	Akhaltsikhe			
	BPAL3015-15	KY086138										*M. interrupta*		Georgia	Akhaltsikhe			
	BPAL3019-15	KY086139										*M. interrupta*		Georgia	Bakuriani			
	BPAL3020-15	KY086140										*M. interrupta*		Georgia	Bakuriani			
AF187809	GBLN0103-06	AF187809										*M. interrupta*		Russia	NW Caucasus, Arkhyz			
FJ462260	GBLN1848-09	FJ462260										*M. interrupta*		Russia	N. Caucasus Arkhyz			
CCDB-03033 H11	BPAL1710-12	KT874705										*M. interrupta*	27-Jul-2008	Russia	Kuban			1700
LOWAB282-09		KY118821										*M. interrupta*		Turkey	Kachkar Mts			
2005-LOWA-140	LOWA140-06	FJ663792										*M. latonigena*	02-Jul-1999	Russia	Dzhezator	49.483	88.117	1800
2005-LOWA-139	LOWA139-06	FJ663793										*M. latonigena*	02-Jul-1999	Russia	Dzhezator	49.483	88.117	1800
CCDB-17966 A01	BPAL2471-14	KT874714										*M. latonigena*	16-Jun-2001	Russia	Tuva, 30 km N Kyzyl			
CCDB-17966 A02	BPAL2472-14	KT874728										*M. latonigena*	16-Jun-2001	Russia	Tuva, 30 km N Kyzyl			
	BPAL3475-16	KY086169										*M. latonigena*		Russia	Altai			
	BPAL3476-16	KY086170										*M. latonigena*		Russia	Altai			
	BPAL3477-16	KY086171										*M. latonigena*		Russia	Altai			
	BPAL3489-16	KY086177										*M. latonigena*		Russia	Buryatia			
AY346234	GBLN0315-06	AY346234										*M. latonigena*		Russia	Buryatiya			
AY350460	GBLN0319-06	AY350460										*M. latonigena*		Russia	Buryatiya			
AY350461	GBLN0320-06	AY350461										*M. latonigena*		Russia	Buryatiya			
CCDB-17951 A04	BPAL2284-14	KT874704										*M. latonigena*	11-Jun-1997	Russia	Tataurovo			
CCDB-17951 A03	BPAL2283-14	KT874739										*M. latonigena*	11-Jun-1997	Russia	Tataurovo			
CCDB-17950 H08	BPAL2467-14	KT874708										*M. mixta*	25-Jul-2004	Afghanistan	Mt. Yamak			3750
CCDB-17950 H06	BPAL2465-14	KT874719										*M. mixta*	25-Jul-2004	Afghanistan	Mt. Yamak			3750
FJ462242	GBLN1866-09	FJ462242										*M. mixta*		Tajikistan	W. Pamir Rushan			
FJ462256	GBLN1852-09	FJ462256										*M. mixta*		Tajikistan	W. Pamir			

Sample ID	BOLD process ID (COI)	COI-5P Accession	ddRADseq data	ddRADseq lineage	COI lineage	*Wolbachia* infection	BOLD process ID (*Wolbachia*)	WSP Accession	WSP allele	ftsZ Accession	ftsZ allele	Taxon	Collection Date	Country	Exact Site	Lat	Lon	Elevation (m)
CCDB-17951 A12	BPAL2292-14	KT874699										*M. mixta*	01-Jul-1992	Tajikistan	Peter I Range, Ganischou			
CCDB-17966 B04	BPAL2486-14	KT874711										*M. mixta*	12-Jul-2004	Tajikistan	Peter I Range			
CCDB-17966 D03	BPAL2509-14	KT874722										*M. mixta*	05-Jul-1994	Tajikistan	Pendzhikent Farob			
CCDB-17966 B06	BPAL2488-14	KT874731										*M. mixta*	19-Jun-2004	Tajikistan	Peter I Range			
CCDB-17949 E02	BPAL2235-13	KT874697										*M. montium*	22-Jun-2013	Israel	Mt. Hermon	33.3127	35.795	2056
CCDB-17949 E03	BPAL2236-13	KT874736										*M. montium*	22-Jun-2013	Israel	Mt. Hermon	33.3127	35.795	2056
		FJ462273										*M. persea*		Iran	Ardabil			
		AF187796										*M. persea*		Lebanon	Les Cedres, Mohafazat Beharre´			
		HQ004810										*M. phoebe*		Romania				
52025		KT792906										*M. phoebe*	5 July 2014	Russia	Samara obl., Kinel district	53°0'13.8	50°9'24.6	
160017		KT792907										*M. phoebe*	27 June 2014	Russia	Samara obl., Bachilova polyana	53°4'28.6	49°6'61.8	
		FJ462281										*M. saxatilis*		Iran	Tehran			
FJ462254	GBLN1854-09	FJ462254										*M. sutschana*		China	Hebei			
AF187805	GBLN0099-06	AF187805										*M. sutschana*		Russia	Chita region, Kyra			
CCDB-17966 G01	BPAL2543-14	KT874696										*M. sutschana*	18-Jun-1995	Russia	Chita reg., Talacha			
CCDB-17966 G03	BPAL2545-14	KT874701										*M. sutschana*	18-Jun-1995	Russia	Chita reg., Talacha			
CCDB-17966 G02	BPAL2544-14	KT874717										*M. sutschana*	18-Jun-1995	Russia	Chita reg., Talacha			
	BPAL2309-14	KY086105										*M. sutschana*		Russia	Primorskij Kray			
CCDB-17949 C05	BPAL2214-13	KT874693										*M. telona*	24-Apr-2013	Israel	Odem Forest	33.174	35.7522	960
CCDB-17968 E09	BPAL2717-14	KT874723										*M. telona*	03-Jul-2014	Israel	Hermon			
CCDB-17949 G08	BPAL2265-13	KT874724										*M. telona*	23-Jun-2013	Israel	Avivim	33.0825	35.4594	616
CCDB-17949 C06	BPAL2215-13	KT874729										*M. telona*	24-Apr-2013	Israel	Odem Forest	33.174	35.7522	960
CCDB-17949 G09	BPAL2266-13	KT874747										*M. telona*	23-Jun-2013	Israel	Avivim	33.0825	35.4594	616
CCDB-17949 G10	BPAL2267-13	KT874749										*M. telona*	23-Jun-2013	Israel	Avivim	33.0825	35.4594	616
		HQ004812										*M. trivia*		Romania				
		HQ004813										*M. trivia*		Romania				
		HQ004814										*M. trivia*		Romania				
		HQ004815										*M. trivia*		Romania				
		HQ004816										*M. trivia*		Romania				
		HQ004817										*M. trivia*		Romania				
		HQ004818										*M. trivia*		Romania				

Table S2. Specimens of *Melitaea didyma* analysed in this study and a summary of the ddRAD data in *de novo* and reference assemblies. Individuals selected for BFD* analyses assuming 5 taxa are marked with *, while individuals selected for analyses assuming 8 taxa are marked with $^{+}$. Specimens selected for *TreeMix* and D-statistics as representatives for each clade are marked with ^{d} in Sample ID.

Clade	Sample ID	Population	Total reads (x10^{6})	***de novo* assembly**				**Reference assembly**				
				Clusters at 90%	Mean depth	Retained loci	Recovered loci	Mapped reads	Clusters total	Clusters depth	Reads consensus	Recovered loci in assembly
**Clade A**	06_M973_RO	Romania	2.28	15274	33.2	5337	524	59642	5845	6.24	1651	350
	07_F189_TR * $^{+}$	Turkey	4.03	24358	55.4	9850	1700	209962	12829	115.8	4437	882
	07_F194_TR * $^{+}$	Turkey	2.48	34519	33.5	11310	1674	247576	16262	53.7	4911	961
	07_F261_TR * $^{+d}$	Turkey	6.02	50392	88.8	14263	1995	244540	27353	26.2	5800	1093
	08_M265_RO * $^{+}$	Romania	2.31	25899	53.8	9138	1664	157324	14989	32.5	4638	886
	11_D714_KZ	Kazakhstan	1.18	17098	16.1	6667	995	68142	9082	22.3	2914	516
	11_G386_KZ * $^{+d}$	Kazakhstan	3.48	47146	18.4	15385	2118	382712	24238	58.8	6136	1138
	11_G595_KZ * $^{+d}$	Kazakhstan	4.06	47318	17.8	15437	2074	398166	23400	54.3	5927	1067
	11_G663_KZ * $^{+d}$	Kazakhstan	3.3	56864	25.8	17012	2318	369878	25657	45.5	5648	1065
	14_E926_SR	Serbia	5.74	25307	18.2	7796	1287	181210	13618	32.5	3276	614
	14_F449_BL	Bulgaria	1.23	16678	24.9	4827	613	123670	9597	53.1	1618	230
	14_F502_GR * $^{+}$	Greece	1.95	27476	26.7	9099	1361	182892	15929	45	4072	676
	14_F516_GR * $^{+}$	Greece	1.48	20478	21.3	7460	1213	172892	13050	50	3709	668
	14_F524_GR	Greece	2.5	16681	29.8	5929	729	124156	9460	62.3	1838	273
	14_F960_GR	Greece	1.88	36357	9.4	8913	1390	195102	20758	33.9	3576	668
	14_G115_GR	Greece	2.21	28807	13.8	8359	1320	194692	18825	30.6	3585	592
	14_G179_GR	Greece	3.27	31195	34.1	9274	1715	236990	19871	22.5	4217	829

Clade	Sample ID	Population	Total reads $(x10^6)$	*de novo* assembly				Reference assembly				
				Clusters at 90%	Mean depth	Retained loci	Recovered loci	Mapped reads	Clusters total	Clusters depth	Reads consensus	Recovered loci in assembly
	14_G632_GR * [+d]	Greece	3.08	58007	14.7	14931	2546	386768	29190	44.7	6029	1152
	14_N358_UA	Ukraine	1.12	31258	11.7	12066	1983	175170	15627	38.2	5076	1039
	14_V093_UA	Ukraine	3.3	32518	40.1	11458	1870	257138	17099	79.4	4724	949
	14_V402_UA	Ukraine	0.12	8381	4.9	4113	814	30494	5878	8.4	1405	274
**Clade B**	08_R519_IT * [+d]	Sicily, Italy	1.36	21986	22.4	8988	2121	147270	10043	62.8	3750	907
	11_H582_IT * [+d]	Sicily, Italy	2.32	23857	60.4	9413	2239	165896	14130	31.6	4453	1149
	11_H748_IT * [+d]	Sicily, Italy	1.39	19218	32.2	7754	1843	124888	10450	42.1	3408	808
	11_H995_IT * [+d]	Sicily, Italy	1.80	40248	17.6	12444	2599	253268	22922	36.0	5456	1310
	11_I076_IT * [+d]	Sicily, Italy	1.19	26924	12.4	8569	1833	165730	14848	32.2	3940	937
**Clade C**	09_X232_FR * [+]	France	1.93	40312	14.7	11869	2476	279780	20151	37.8	5330	1298
	10_A449_FR * [+]	France	0.91	22729	16.8	9457	2197	134320	13104	28.6	4777	1225
	10_A578_FR	France	1.29	29083	16.1	9186	1762	191166	19295	25	4398	1003
	10_B979_FR * [+]	France	2.71	40840	17.7	12316	2402	310712	21028	49.7	5629	1429
	10_B993_FR * [+]	France	2.62	36188	33.3	11311	2372	235530	18688	43.7	5078	1244
	10_C562_IT	Italy	1.77	13311	75.9	4480	725	87592	8896	42.7	1667	273
	11_I119_IT * [+]	Italy	0.95	24702	10.5	8622	1798	128742	15079	22.8	4260	1034
	11_I689_FR * [+d]	France	2.65	39531	24	12549	2834	295120	24756	36.3	6170	1561
	11_I690_FR	France	2.71	35575	26.4	11326	2524	289072	20227	45.2	5449	1370
	11_I691_FR	France	3.46	45263	37.9	12475	2422	272110	23106	46.5	5419	1372

Clade	Sample ID	Population	Total reads (x10^6)	***de novo* assembly**				**Reference assembly**				
				Clusters at 90%	Mean depth	Retained loci	Recovered loci	Mapped reads	Clusters total	Clusters depth	Reads consensus	Recovered loci in assembly
	11_I766_FR	France	3.11	49303	30	12876	2331	383782	23303	33	5081	1201
	11_J146_CH[d]	Switzerland	3.6	49038	42.1	13873	2804	309550	23328	42.3	5552	1394
	11_J199_FR	France	1.74	28745	30.1	10340	2252	188032	15267	35.5	4729	1111
	11_J622_IT *[+]	Italy	3.62	38588	38.7	11996	2410	283910	19980	88.2	4652	1129
	12_O043_IT *[+]	Italy	5.95	130628	16.4	26196	2717	706154	64475	34.3	5970	1324
	12_O695_FR	France	1.87	34320	20.6	11375	2683	230602	21511	32.3	5665	1501
	12_O756_FR	France	3.8	41203	55.8	11642	2331	220854	23501	26.7	5033	1266
	12_O852_FR[d]	France	3.06	49632	20.6	13744	2914	373276	26048	40.9	6389	1618
	12_O903_IT	Italy	1.3	26338	22.4	9553	2103	154660	15999	26.3	4552	1083
	12_O967_FR	France	2.31	33119	32.7	10920	2155	187056	17017	32.9	5009	1271
	12_P518_FR	France	2.63	38884	18.9	11638	2281	314420	17962	79.3	4889	1163
	12_P593_FR	France	1.64	25433	30	9273	1973	147616	15834	31.4	4447	1071
	12_P738_FR	France	1.52	22080	18.9	7857	1650	171090	15531	35.1	4083	939
	12_Q005_FR	France	1.49	21618	31.3	7789	1806	173616	16320	45.8	4201	1018
	12_Q067_FR	France	1.2	22320	17.8	8259	1788	168776	16449	31.1	4214	930
	12_Q108_FR	France	1.82	28594	22.4	9660	2284	216026	21778	30	5176	1299
	12_Q313_FR	France	1	23929	13.2	8850	1970	154020	16189	27.1	4599	1139
	12_Q342_FR	France	2.71	25984	44.5	9477	2154	230218	15571	34	4725	1225
	12_Q922_IT	Italy	3.36	22750	23.6	7917	1584	184080	14917	47.9	3816	821

Clade	Sample ID	Population	Total reads $(x10^{6})$	*de novo* assembly				Reference assembly				
				Clusters at 90%	Mean depth	Retained loci	Recovered loci	Mapped reads	Clusters total	Clusters depth	Reads consensus	Recovered loci in assembly
	12_R197_IT * [+]	Italy	2.23	33055	25.6	12192	2722	247562	19296	44.3	5692	1454
	12_R360_IT	Italy	3.62	28497	39.6	8777	1536	168472	12917	40.7	3416	750
	13_S727_IT * [+]	Italy	2.19	26052	35.2	9885	2180	175724	14622	51.2	4248	1010
	13_U302_IT	Italy	5.01	42209	48.4	11070	1699	195064	19359	33.9	3465	719
	14_A356_IT	Italy	2.33	28158	43.3	9196	1814	182500	16071	44.3	4031	909
	14_H910_FR [d]	France	2.32	44470	19.8	13097	2940	270434	27814	25.5	5913	1449
	14_N085_IT	Italy	1.29	20529	16.6	7421	1664	145756	12681	41.2	3746	883
	14_W669_IT	Italy	2.61	30856	35.9	10658	2121	217732	16638	49.9	4496	1081
	14_W858_IT	Italy	2.84	22778	62.2	8825	1848	175536	11864	61.4	3479	827
	15_H077_IT	Italy	2.24	31959	26	10311	2007	210362	17707	39.1	4603	1081
	15_J468_FR	France	1.35	26273	12.4	9251	2118	170442	18686	28.8	4817	1133
	15_K607_IT	Italy	2.03	28905	23.9	10192	2245	216260	17364	44.3	5020	1252
	15_L153_IT	Italy	2.05	26591	31.4	9543	1969	183886	14037	48.1	4442	1060
	15_L572_IT [d]	Italy	3.53	48735	41.9	13524	2818	328446	24536	49.1	5787	1457
	15_M034_IT	Italy	3.24	44554	28.7	13189	2786	365898	26581	42.3	6022	1479
	15_M518_IT	Italy	2.64	31180	37.1	10033	2073	233116	17417	66	4756	1165
**Clade D**	06_A056_MO	Morocco	1.19	19869	21.4	6923	1472	125092	11244	40.8	3272	739
	06_G603_MO * [+d]	Morocco	2.83	29637	33.2	7816	1502	264250	15270	82.6	3334	700
	11_F172_MO * [+d]	Morocco	3.09	36496	19.7	12968	2281	360028	24329	64.1	5824	1361

Clade	Sample ID	Population	Total reads (x10^6)	***de novo* assembly**				**Reference assembly**				
				Clusters at 90%	Mean depth	Retained loci	Recovered loci	Mapped reads	Clusters total	Clusters depth	Reads consensus	Recovered loci in assembly
	11_F879_MO * [+ d]	Morocco	3.14	39557	21.1	13140	2272	388136	24631	55.9	5805	1331
	12_L827_MO * [+ d]	Morocco	2.45	39441	16.6	13409	2250	351008	20860	51.2	5547	1190
	12_N510_TN * [+ d]	Tunisia	1.90	38983	18.9	12627	2395	248060	22387	37.5	5724	1373
	12_R298_MO	Morocco	1.76	15547	27.9	6232	984	145658	10280	55.9	2822	523
	13_S473_MO	Morocco	1.90	21259	21.7	8169	1497	166364	11948	69.8	3680	849
	15_Q052_AL	Algeria	0.26	10845	9.5	5074	1103	56032	8201	12.7	2614	555
**Clade E**	08_J709_SP * [+ d]	Spain	2.13	57522	12.3	13094	2529	368606	24180	40.0	5683	1366
	08_J735_SP * [+ d]	Spain	4.40	93052	12.8	19474	2328	584200	32936	38.4	5090	1100
	08_J737_SP * [+]	Spain	2.67	40706	17.4	12095	2235	359580	22230	54.6	5355	1287
	08_J742_SP * [+]	Spain	2.21	45260	13.8	13269	2241	319584	22549	41.3	5313	1293
	08_J993_SP * [+ d]	Spain	2.59	44521	19.5	12803	2371	372706	20395	57.7	5112	1153
	08_L915_SP	Spain	2.36	32009	20.1	10440	1970	288670	17769	50.5	4825	1158
	08_L951_SP	Spain	2.05	27384	19.2	9816	1874	257652	17286	51.4	5034	1209
	08_P048_SP [+]	Spain	1.81	24840	34.1	9532	2113	178930	15001	40.5	5037	1247
	08_P370_SP [+ d]	Spain	2.51	45280	23.6	12985	2286	304924	22921	35.8	5266	1262
	09_V584_SP	Spain	2.64	42804	17.9	12371	2167	300940	22653	44.2	5492	1364
	11_D602_SP	Spain	2.11	32095	20.1	10542	1861	241174	21479	37.9	4827	1117
	11_J247_SP [+]	Spain	3.07	41516	45.6	11579	2259	235334	18293	27.5	4688	1139
	14_V970_SP [+ d]	Spain	2.32	41624	21.9	13118	2590	292324	19658	53.7	5506	1367

Clade	Sample ID	Population	Total reads (x10^6)	*de novo* assembly				Reference assembly				
				Clusters at 90%	Mean depth	Retained loci	Recovered loci	Mapped reads	Clusters total	Clusters depth	Reads consensus	Recovered loci in assembly
Outgroup	14_I563_IT * [+ d]	Italy	0.99	45229	63.2	12810	844	30955	4981	73.82	1940	131
	15_A753_PT * [+ d]	Portugal	2.34	41414	90.4	10153	4057	113910	15916	13.74	5315	200
		**AVERAGE**	**2.42**	**34168**	**28.3**	**10634**	**2003**	**238205**	**18636**	**43.5**	**4623**	**1059**

Table S3. Summary of ddRAD and mtDNA data sets.

**Data source**	**ddRAD**			**mtDNA**
Data matrix	ddRAD_mt_m4	ddRAD_dn_out	ddRAD_ref	mtDNA_COI
Outgroup	NA	14_I563_IT, 15_A753_PT	14_I563_IT, 15_A753_PT	14_I563_IT, 15_A753_PT
Assembly method	de novo – reference [a]	de novo	Reference [b]	NA
Number of taxa	92 [c]	95	95	93
Number of loci	22,353	22,342	14,525	1
Alignment length (bp)	3,489,654	3,487,180	2,548,000	658
SNPs	143,201	144,004	116,942	141
Informative sites	46,371	46,096	41,362	88
Missing data (%)	90.9	91.3	89.7	0

[a] *Melitaea cinxia* mitochondrion genome (CM002851) was used as reference genome.
[b] *Melitaea cinxia* whole genome sequences (GCA_000716385) were used as reference.
[c] Specimen “RV-06-M973” was removed due to the low number of recovered loci in the final data matrix.

Table S4. Primers and PCR protocols used for the amplification of COI, wsp and ftsZ.

COI

Reagents	µl per reaction
Buffer 5X	5
MgCl2 (25 mM)	2
dNTPs (10mM)	0.5
LepF1 (10µM)	0.5
LepR1 (10µM)	0.5
H2O	14.4
Taq polymerase (Promega)	0.1
DNA extraction	2

Total volume = 25

PCR program

Step	Temperature	Duration	Cycles
1	92ºC	60 s	
2	92ºC	15 s	Steps 2-4, 5X
3	48ºC	45 s	
4	62ºC	150 s	
5	92ºC	15 s	Steps 5-7, 35X
6	52ºC	45 s	
7	62ºC	150 s	
8	62ºC	420 s	

wsp

Reagents	µl per reaction
Buffer 10X	1
MgCl2 (25 mM)	1
dNTPs (10mM)	0.2
wsp81_F (10µM)	0.5
wsp691_R (10µM)	0.5
H2O	5.7
AmpliTaq Gold (Applied Biosystems)	0.1
DNA extraction	1

Total volume = 10

PCR program

Step	Temperature	Duration	Cycles
1	95ºC	300 s	
2	95ºC	30 s	Steps 2-4, 38X
3	55ºC	30 s	
4	72ºC	120 s	
5	72ºC	120 s	

ftsZ

Reagents	µl per reaction
Buffer 10X	1
MgCl2 (25 mM)	1
dNTPs (10mM)	0.2
ftsZ_F (10µM)	0.5
ftsZ_R (10µM)	0.5
H2O	5.7
AmpliTaq Gold (Applied Biosystems)	0.1
DNA extraction	1

Total volume = 10

PCR program

Step	Temperature	Duration	Cycles
1	95ºC	300 s	
2	95ºC	30 s	Steps 2-4, 38X
3	54ºC	30 s	
4	72ºC	120 s	
5	72ºC	120 s	

Primer name	Primer sequence (5´ - 3´)	Direction	Marker	Reference
LepF1	ATTCAACCAATCATAAAGATATTGG	Forward	COI	Hebert et al. 2004
LepR1	TAAACTTCTGGATGTCCAAAAAATCA	Reverse	COI	Hebert et al. 2004
wsp81_F	TGGTCCAATAAGTGATGAAGAAAC	Forward	wsp	Baldo et al. 2006
wsp691_R	AAAAATTAAACGCTACTCCA	Reverse	wsp	Baldo et al. 2006
ftsZ_F	ATYATGGARCATATAAARGATAG	Forward	ftsZ	Baldo et al. 2006
ftsZ_R	TCRAGYAATGGATTRGATAT	Reverse	ftsZ	Baldo et al. 2006

References

Baldo L, Dunning Hotopp JC, Jolley KA, Bordenstein SR, Biber SA, Choudhury RR, Hayashi C, Maiden MC, Tettelin H, Werren JH. 2006. Multilocus sequence typing system for the endosymbiont *Wolbachia pipientis*. *Applied and Environmental Microbiology* **72**, 7098–7110. (doi:10.1128/AEM.00731-06)

Hebert PDN, Penton EH, Burns JM, Janzen DH, Hallwachs W. 2004. Ten species in one: DNA barcoding reveals cryptic species in the neotropical skipper butterfly *Astraptes fulgerator*. *Proc. Natl. Acad. Sci. USA* **101**, 14812–14817. (doi:10.1073/pnas.0406166101)

Table S5. Minimum p-distance matrix between ten COI lineages of *M. didyma*. Minimum and maximum values are in blue and red, respectively.

	Minimum p-distance (%)									
	**L1**	**L2**	**L3**	**L4**	**L5**	**L6**	**L7**	**L8**	**L9**	**L10**
**L1**	n/a									
**L2**	2.43	n/a								
**L3**	2.43	2.43	n/a							
**L4**	2.89	3.04	1.82	n/a						
**L5**	2.28	3.04	2.43	2.43	n/a					
**L6**	3.34	3.34	3.34	3.65	2.89	n/a				
**L7**	3.34	3.34	3.04	3.65	2.74	1.52	n/a			
**L8**	4.26	5.02	4.41	3.8	3.8	3.95	3.8	n/a		
**L9**	3.65	4.26	3.50	3.19	2.58	2.74	2.13	1.22	n/a	
**L10**	5.93	7.45	6.53	6.23	5.62	6.08	6.69	7.14	6.38	n/a

Table S6. Pairwise F_{ST} values among all clades of *M. didyma* using unlinked SNP data. An asterisk denotes significant statistical support from 1,000 permutations ($p < 0.05$).

	A	B	C	D	E	Outgroup
A	–					
B	0.79959 *	–				
C	0.63294 *	0.95894 *	–			
D	0.68593 *	1.00000 *	0.88916 *	–		
E	0.67690 *	0.87873 *	0.65320	0.65689 *	–	
Outgroup	0.61531 *	1.00000 *	0.94708 *	1.00000 *	0.87171 *	–

Table S7. Species delimitation scenarios for *Melitaea didyma*. a) Results assuming five species. b) Results assuming eight species. The table shows the different species delimitation models for the group evaluated with the BFD* method and the associated results. Each row indicates a different species delimitation model and two samples ('14_I563_IT' and '15_A753_PT') were included in all tests as outgroup taxa (O). The best delimitation scenario is shown in bold.

a)

Scenarios	Description	n species (w/o outgroup)	MLE	Bayes factor	Rank
Current taxonomy	(A,B,C,D,E),(O)	1	-3783.7	NA	9
Split 2.1	(A,B,C),(D,E),(O)	2	-3718.1	-131.2	7
Split 2.2	(A),(B,C,D,E),(O)	2	-3750.6	-66.2	8
Split 2.3	(A,C,D,E),(B),(O)	2	-3686.4	-194.5	6
Split 3.1	(A),(B,C),(D,E),(O)	3	-3661.3	-244.9	3
Split 3.2	(A,B),(C),(D,E),(O)	3	-3678.8	-209.7	5
Split 3.3	(A,B,C),(D),(E),(O)	3	-3667.4	-232.6	4
Split 4	(A),(B,C),(D),(E),(O)	4	-3612.1	-343.3	2
**Split 5**	**(A),(B),(C),(D),(E),(O)**	**5**	**-3551.0**	**-465.3**	**1**

b)

Scenarios	Description	n species (w/o outgroup)	MLE	Bayes factor	Rank
Current taxonomy	(A,B,C,D,E),(O)	1	-224334.8	NA	10
Split 2.1	(A,B,C),(D,E),(O)	2	-213984.6	-20700.5	7
Split 2.2	(A),(B,C,D,E),(O)	2	-215945.4	-16778.8	9
Split 2.3	(A,C,D,E),(B),(O)	2	-215330.0	-18009.7	8
Split 3.1	(A),(B,C),(D,E),(O)	3	-207846.9	-32975.8	4
Split 3.2	(A,B),(C),(D,E),(O)	3	-208976.7	-30716.1	5
Split 3.3	(A,B,C),(D),(E),(O)	3	-212030.2	-24609.2	6
Split 4	(A),(B,C),(D),(E),(O)	4	-205842.8	-36984.1	3
Split 5	(A),(B),(C),(D),(E),(O)	5	-199276.4	-50116.7	2
**Split 8**	**(A_1),(A_2),(B),(C_1),(C_2),(D),(E_1),(E_2),(O)**	**8**	**-198600.0**	**-51469.5**	**1**

Table S8. Tests of admixture using five clades and 25 specimens of *M. didyma* (five for each clade), as well as two specimens (*M. trivia* and *M. deione*) used as outgroup. P1, P2, and P3 indicate species/population used in a given topology position when testing for admixture using Patterson's D-statistics. Each test was repeated over all possible four-sample replicates (n), with a range of Z-scores reported, and the number of significant replicates shown (nSig). Outgroup, not shown in the table, consists of two individuals ('14_I563_IT' and '15_A753_PT'). n loci used is the number of loci analyzed in each test.

Test	P1	P2	P3	Range Z	nSig/n	nSig/n (%)	n loci used
1	A	A	B	(0.2 – 5.4)	2/49	4.1	104
2	A	A	C	(0.0 – 9.5)	6/49	12.2	110
3	A	A	D	(0.0 – 11.8)	12/49	24.5	106
4	A	A	E	(0.1 – 3.4)	0/49	0	108
5	B	B	A	(0.2 – 8.3)	4/49	8.2	114
6	B	B	C	(0.0 – 23.5)	8/49	16.3	124
7	B	B	D	(0.0 – 42.4)	9/49	18.4	117
8	B	B	E	(0.0 – 5.0)	2/49	4.1	118
9	C	C	A	(0.0 – 12.6)	10/49	20.4	117
10	C	C	B	(0.0 – 45.5)	12/49	18.4	122
11	C	C	D	(0.0 – 29.0)	12/49	18.4	120
12	C	C	E	(0.1 – 4.9)	2/49	4.1	125
13	D	D	A	(0.0 – 10.5)	9/49	18.4	117
14	D	D	B	(0.0 – 91.2)	10/49	20.4	118
15	D	D	C	(0.0 – 12.0)	7/49	14.3	124
16	D	D	E	(0.0 – 2.2)	0/49	0	128
17	E	E	A	(0.0 – 5.8)	5/49	10.2	114
18	E	E	B	(0.0 – 12.3)	9/49	18.4	114
19	E	E	C	(0.0 – 9.1)	13/49	25.5	123
20	E	E	D	(0.0 – 3.6)	0/49	0	122
